# “I like the way I am, but I feel like I could get a little bit bigger”: Perceptions of body image among adolescents and youth living with HIV in Durban, South Africa

**DOI:** 10.1371/journal.pone.0227583

**Published:** 2020-01-10

**Authors:** Patrick Nyamaruze, Kaymarlin Govender

**Affiliations:** 1 The Discipline of Psychology, School of Applied Human Sciences, University of KwaZulu-Natal, Durban, South Africa; 2 Health Economics and HIV and AIDS Research Division, University of KwaZulu-Natal, Durban, South Africa; Centre for Sexual Health & HIV/AIDS Research, ZIMBABWE

## Abstract

Body image concerns are common among people living with HIV (PLHIV). Research into how young people living with HIV (YPLHIV) experience and make sense of feared or actual body changes is limited, yet these changes have emotional, psychological and interpersonal implications for young people who associate physical attractiveness with social desirability. The current study examined the subjective perceptions of body appearance and coping mechanisms among a sample of YPLHIV in Durban, South Africa. An interpretive qualitative inquiry was adopted to understand their lived experiences in relation to their body image and body satisfaction. In-depth interviews were conducted with 18 YPLHIV (15–24 years). Findings indicate physical and psychosocial effects of living with HIV among young people including weight loss, skin sores, body dissatisfaction, loss of self-esteem and social withdrawal. The study builds on previous research suggesting that PLHIV may experience a discrepancy between their actual self and ideal self. Enhancing existing coping mechanisms such as religious beliefs, support networks and physical exercises among YPLHIV can counter the physical and psychosocial effects of living with HIV and improve well-being. Body image concerns should be acknowledged when addressing HIV-related health in both health and family settings.

## Introduction

The introduction and wide availability of antiretroviral therapy (ART) has offered hope to people living with HIV (PLHIV) as the treatment has reduced mortality, increased life expectancy and improved quality of life significantly [1]. As more HIV-positive young people live longer, focus is slowly shifting to improving their health-related quality of life. Growing up with HIV is still challenging in various aspects [2]. One of the challenges faced by young people living with HIV (YPLHIV) is managing debilitating physical and psychosocial factors arising from living with the condition itself and its treatment [3, 4], with HIV infected adolescents usually showing stunted and slow growth, compared to their uninfected counterparts [5]. Studies with both male and female participants have shown that alterations in body appearances have significant effects on the psychosocial well-being and quality of life of PLHIV [6, 7]. Growth retardation in all forms has a negative psychological impact on adolescents for whom body image is an integral part of the adolescence phase [8]. There is limited research on ART-related lipodystrophy (a disorder of fatty tissue characterized by a selective loss of body fat) in low- and middle-income countries especially among adolescents [9], yet the prevalence of lipodystrophy is substantial in these setting [10].

Body image relates to a person’s perceptions, feelings and thoughts about their body [11]. However, these feelings and thoughts are not entirely inherent but rather, the image that an individual has of their body is largely determined by social experience [12]. Entrenchment of an appearance-oriented culture reinforced by significant others in the adolescents’ immediate environments, including family, peers, and romantic partners [13] ignites an increased evaluation and focus on body appearance. Experiences of body evaluation for YPLHIV are complicated by notions of living with such a stigmatized condition [14].

Our review of literature found very little research on perceptions and experiences of body image among YPLHIV. Studies investigating body image and changes in PLHIV have been predominantly conducted among men who have sex with men [15, 16, 17]. Other qualitative studies with HIV-positive men and women found that patients’ current perceptions of their body image were less favorable than perceptions of themselves before they were infected [11]. A diagnosis of AIDS was significantly related to poorer quality of body image and lowered life satisfaction [18]. A closely related concept to body image is body image satisfaction, which has been found to be low in PLHIV as compared to HIV-negative individuals, resulting from deterioration in their body shape, size and function [4, 17, 19].

Issues pertaining to body image have emotional, psychological and interpersonal implications for young people who associate attractiveness with social desirability, yet these issues have not been adequately researched in the African context among adolescent population groups [11, 19, 20]. Furthermore, studies have often been conducted in clinical settings and from a quantitative approach. In light of these limitations, we undertook a qualitative study to understand perceptions of YPLHIV pertaining body image experiences. Here, we describe the subjective perceptions of body appearance and coping mechanisms among a sample of YPLHIV. We used self-discrepancy theory to investigate young people’s perceptions of their body appearance, their level of satisfaction with their body image and the psychosocial implications emanating from own evaluation of body appearance.

## Theoretical framework

Self-discrepancy theory (SDT) explores three domains of self [21]. The ‘actual’ self, which reflects an individual’s perceptions of their own attributes or characteristics, is referenced in relation to the ‘ideal’ self and the ‘ought’ self. Research using nonclinical samples has mostly supported associations between actual/ideal discrepancies (as opposed to the actual or ought self) [22]. A key feature of the SDT is to outline the specific emotional consequences of perceiving a discrepancy between dimensions of selves [23]. The relevance of this theory to the concept of body image is largely based on the existence of cultural norms that espouse particular standards of attractiveness. Inconsistencies in self-states lead to psychological discomfort and negative emotions [24] which in turn, reinforce poor self-appraisals. Research examining self-discrepancies in the context of body image indicates that females generally experience more body image discrepancy than their male counterparts; however, there has been increasing awareness of the importance of body image among males [23].

## Methodology

An interpretive qualitative inquiry was adopted to understand the lived experiences of YPLHIV in relation to their body image and body satisfaction. Data were collected through in-depth interviews with YPLHIV August to October 2018. The study was conducted in Durban, South Africa in a youth friendly centre where the respondents collected their treatment and received social support. In line with the purposive sampling technique, individuals with special knowledge and attributes [25]: aged 15–24 years, living with HIV, receiving antiretroviral drugs (ARVs) and, reporting knowledge of mode of transmission, were recruited. With the assistance of clinicians, 20 YPLHIV were selected and requested to participate in the study. Among the young people approached, two could not participate because of time constraints as they had to attend classes and the researcher could not schedule an appointment with them since they did not stay close to the centre. The final sample comprised 18 participants.

Interviews were conducted in English and isiZulu in a quiet office at the centre which provided a confidential atmosphere in which respondents could share sensitive and personal information. The researcher broadly explained the purpose of the study as focusing on psychosocial challenges that YPLHIV may experience and enquiries pertaining to body image emerged gradually as the interviews progressed.

Of the 18 interviews conducted, 12 were single session interviews. Two repeat interviews were conducted with five respondents. Only one respondent was interviewed three times. The follow-up interviews provided the opportunity not just to verify understandings formed in the first interview but to also seek clarification on things that the researcher had not fully understood. Subsequent interviews were tailored to individuals, based on what was said in the previous interviews [26]. Discussions were informed by an interview guide which was primarily centered on the thoughts, experiences and feelings of respondents living with HIV, a chronic condition, and its implications for evaluations of body image.

Interviews were conducted within three months, with most of the repeat interviews conducted three weeks after the initial ones. Interviews were audio-recorded with permission from respondents. Interview duration was 35–50 minutes. Audio-recordings were transcribed non-verbatim and translated to English, thus some repeated words that did not contribute to the underlying message were removed. Initially, NVivo 10 software program (QSR International, Melbourne, Australia) was used to sort and organize the large data set. Thereafter, the six steps of thematic analysis were used to further analyze the data and reach conclusions [27]. The first author conducted all analyses and utilized peer debriefing to enhance the trustworthiness and credibility of the analysis [28].

## Ethical considerations

The Human and Social Sciences Research Ethics Committee of the University of KwaZulu- Natal approved this study (#HSS/0522/018D). Ethical considerations of voluntary participation, informed consent, and confidentiality were observed. Informed consent (18 years or older) and parental consent along with adolescent assent (age 15–17 years) were obtained verbally and in written form from all respondents prior to the interviews. Respondents’ right to withdraw from the study at any point without negative consequences for making such a decision was explained prior to the study. To protect the identity of respondents, pseudonyms are used to report findings.

## Results

We conducted 18 in-depth interviews with 18 YPLHIV (n = 8 male; n = 10 female). Of these 8 reported vertical HIV infection and 10 reported acquiring the condition horizontally. Respondents provided various descriptions of their body image. The common descriptions were related to physical attributes that included weight loss and skin sores. Physical attributes as a result of their chronic condition and its treatment were described in conjunction with perceptions of body satisfaction and psychological and social sequelae. These findings are described in detail below.

### Body appearance concerns

Participant responses alluded to body dissatisfaction arising mostly from weight loss and the presence of skin sores. Among males, there was a desire to gain some weight and have a bigger body size that corresponded to becoming a young adult. One of the responses indicating weight concerns in males is indicated below;

I like the way I am, but I feel like I could get a little bit bigger instead of remaining too small you see, just to gain a little bit in my body, so I used to get sick, lose weight and gain it again, so that’s how it is

(Kagiso; Male, 20 years old).

Among the female respondents, weight loss was associated with a desire to gain weight in certain body areas. Having a, “*bigger bum or being two sizes bigger or a size bigger at least”* was deemed important to enhance their looks and perceived physical attractiveness *(Ayanda; Female*, *21 years old)*.

Respondents struggled to maintain constant body weight as it was constantly changing because of periods of sickness likely caused by non-adherence to treatment as well as use of new treatment regimes. In relation to this issue the respondents explained that;

At first I didn’t take them [ARVs] on time and instead of getting better it was getting worse and my body was not gaining and then she [nurse] told me I must always take them because she gave me like a timetable indicating when I should take them and I started getting used to it and I got back to normal

(Ayanda; Female, 21 years old).

There appeared to be discrepancies in how respondents felt and thought about their body as compared to their actual body weight. They thought that they had lost significant amount of weight. However, feedback from others revealed that they *“looked the same” (Sfiso; Male*, *19 years old)*.

Perceptions of body image, particularly among female respondents were not solely limited to the physical build of the body but were also influenced by the skin condition. A few of the female respondents indicated the presence of sores on their skin resulting from their HIV seropositive status and spots on the skin which were a reminder of previous sores. As a result, respondents were uncomfortable showing off their skin, hence they made efforts to cover those parts of their body that bore spots by always wearing *“long jeans*, *if not pants it’s leggings” (Thandie; Female*, *22 years old)*.

However, a few of the respondents, particularly those who self-reported horizontal infection reported that they were satisfied with their body image. The ability to perform any duties without being physically constrained in any way contributed to body satisfaction. One respondent explained, “*I am satisfied because I can do things that I want to do and my body is flexible and allows me to do those things” (Buhle; Female*, *21 years old)*. In one instance, body satisfaction was explained in terms of “normalcy” as the perceived body image was not different from significant others and peers, hence it was embraced without any fear of shame.

Regularly eating food with nutritional value and taking treatment as prescribed led to better health outcomes and respondents were more likely to have confidence in their body image.

My body is okay, I don’t have any problems, I have a rich body and I am okay. So, they ask me what is it that I am eating that makes me gain so much weight that is why I am saying even the pills do not affect me and I also think I am eating what is right and I must continue with that

(Zanele; Female, 22 years old).

Having a healthy and fit body is important for PLHIV, particularly those on treatment, as this does not differentiate them, at least in a perceptual sense, from the rest of the population.

### Psychological and social effects

Positive body image and healthy self-esteem are important to young people’s health and well-being. Participant responses indicated that negative feedback on body appearance from both peers and significant others impacted on their self-esteem and confidence as narrated below;

You see, I have been sick and lost weight, that’s where I started lacking self-love…Let me say maybe I have lost weight and end up wearing size 28, since I am size 30, now that is where I lack self-love. If a person says I am thin, I always remember that indeed I have lost weight

(Philani; Male, 17 years old).

A wasting body and allied weight loss were associated with loss of self-confidence and self-esteem.

I end up undermining myself, and when I look at myself I feel like I have lost too much weight, especially if I have been sick and lost weight that is where I undermine myself and feel like I do not like myself, except when I gain, you see right now I can feel myself [self-confident]

(Linda; Female, 20 years old)

Respondents also expressed worry and apprehension regarding making impression and initial contact with potential partners. The fear that partners may notice some flaws within their body made them uneasy as they did not know, “*how the person will respond” (Mandla; Male*, *16 years old)*.

The constant questions about one’s weight loss served as a constant reminder making the individual aware of their current state. This caused distress as explained by one of the respondents that, *“Before*, *I used to pretend like it doesn’t make me feel… you know like it doesn’t eat me*. *I just felt down and would go home and then think by myself what happened to me” (Langa; Male*, *23 years old)*.

### Coping mechanisms

Physical changes explained by respondents were commonly associated with psychosocial implications like anxiety, feelings of isolation and inferiority. However, the respondents described the various mechanisms and resources they employed to cope, including belief in God, engaging in sporting activities, emotional detachment and social support. Respondents mentioned that during times of difficulty, prayers and belief in God kept them strong and resilient. For others, engaging in sporting activities gave them a worthwhile activity which also contributed to their physical well-being and nurtured a sense of belonging. The common sports/physical exercises mentioned by respondents were soccer, netball and jogging. Social support from friends, romantic partners and family in the form of unconditional love and health tips were identified by most of the respondents as crucial in coping with the various stressors of living with HIV.

## Discussion

Overall, our findings indicate physical and psychosocial effects of living with HIV among young people including weight loss, skin sores, body dissatisfaction, loss of self-esteem and social withdrawal. The body is inextricably tied to HIV/AIDS since it is through the body that people are exposed to the infection and also experience the condition. Both male and female respondents raised concerns over unintentional weight loss. One of the salient findings is that although both males and females cited weight loss as a problem, this was experienced differently. In males, concern focused on a small body size that did not correspond to the age of the respondents. Male respondents were self-conscious and ashamed about not being physically developed as compared to their peers. This desire for a bigger body size highlights the need to achieve masculine features synonymous with adolescence [29]. Young males living with HIV may be more distressed about noticeable maturational delay than about their underlying chronic condition. Those with stunted growth may need psychological and nutritional support to achieve optimum growth.

Lack of a big “bum” was a concern particularly for females as it impacted on their feelings and perceptions of being “sexy”. This ‘ideal’ body type is prominent in similar settings and includes being shapely and curvy [30]. In this setting, desire to achieve a certain body weight was influenced by cultural perceptions of beauty and partner preferences. Cultural norms and standards of beauty appear to be largely responsible for perpetuating notions of physical attractiveness [31, 32]. The imposition of these norms regarding socially acceptable appearance and body build can shape one’s personal goals for appearance. In this setting, this can be seen through the desire to be thick and curvaceous by the female respondents and to be bigger and muscular by the male respondents. However, when they believe that they are not living up to these ideals, they experience emotional distress such as shame and self-doubt. Self-discrepancy theory predicts actual versus ideal self-discrepancy to be associated with dissatisfaction with the self that manifest as shame [21] and body dissatisfaction [23]. Accordingly, interventions to change body image discrepancy may be targeted at changing unrealistic and unattainable internalized self-guides imposed by culture.

Body satisfaction was commonly reported by those respondents who had managed to maintain an average body weight and who felt physically strong, as this eradicated feelings of being different. This was in contrast to those who expressed dissatisfaction with their bodies and wished to gain overall body weight, with some wanting to gain flesh in specific body parts whilst others wanting to get rid of skin spots which were a reminder of a prior skin condition. Other researchers also found that dissatisfaction with physical appearance was a common experience for many YPLHIV [19]. Interestingly, respondents who indicated perinatal mode of infection reported greater body dissatisfaction. One possible reason for this is that perinatally infected adolescents experience visible growth and pubertal delays that distinguish them from their uninfected peers and those horizontally infected [33, 34]. Perinatally infected young people may experience more body dissatisfaction because of longer period of living with HIV infection, suboptimal adherence and treatment failure [35].

Mention of HIV-associated skin conditions was common among female respondents and associated with a variety of negative psychosocial impacts. These included feelings of discomfort in social situations, unsolicited questions from romantic partners about one’s skin and attempts at concealing sores and spots by wearing clothes that cover the whole body. Concealment of the body to manage other people’s awareness of an underlying condition has also been reported in other chronic conditions like cancer [36]. In a society that emphasizes smooth skin in females [37], skin sores can be a source of shame especially when individuals compare their actual and ideal self and find a discrepancy. A combination of skin condition treatment and cognitive mechanisms that emphasize acceptance and acknowledgment of the existence of spots as part of one’s being can lead to positive psychological effects and body image satisfaction.

Respondents’ narratives indicated that they supplemented their diet and engaged in various physical activities such as playing soccer and netball as a way of keeping fit and regaining control of their physical capabilities. Similarly, a study on muscular strength training with PLHIV showed not only an improvement in physical function and well-being, but also psychosocial significance of avoiding being stigmatized [38]. Furthermore, engaging in sporting activities fostered feelings of belonging to a community. The findings highlight the benefits of encouraging YPLHIV to engage in sport and physical exercise as it impacts positively on physical health and consequently body image and overall well-being.

It emerged that YPLHIV experience significant psychological challenges including anxiety, worry, and stigma which, if not addressed, often lead to self-isolation and avoidance of proactive behaviors [39]. Support from friends, family and intimate partners was frequently mentioned as a strategy of counteracting the psychosocial challenges experienced by YPLHIV. YPLHIV spoke positively of the support and love (emotional support) they received from both family and friends. In instances where the individual had lost some weight or the skin had spots, they were encouraged to try different food choices and apply certain lotions (informational support). Knowing that they had people who gave them positive feedback (appraisal support) and encouraged them to look good boosted their perceptions of body image. Body image is elastic and changeable through new information and social experience [40]. Thus, positive feedback from family and peers can enhance development of a positive and healthy body image.

While this study highlighted the physical and psychosocial experiences of YPLHIV as well as the coping mechanisms utilized, it had some limitations. These include the cross-sectional nature of the study and use of a single research site. Further, the sample size was relatively small, rendering it difficult to generalize study findings. Future research is needed to longitudinally explore patterns of change in body image among a broader sample of YPLHIV as adolescents’ relationship to their bodies are likely to be fluid and changeable as they progress through adolescence. Despite these limitations, we conducted more than one in-depth interview with some of the respondents which allowed us to extensively explore the respondents’ feelings and perspectives on body image. The follow-up one-on-one interviews were productive as each respondent had enough time and opportunity to share his/her feelings and experiences.

## Conclusion

YPLHIV are not a homogenous group as they experience physical changes in the form of weight loss and body sores differently, which may lead to body image dissatisfaction. Interventions should be cognizant of this diversity and be tailor made to suit the unique needs of YPLHIV. The body image perceptions and experiences of YPLHIV are not fixed but rather dynamic and changeable, and require ongoing discussion and attention as young people continually negotiate their identity. Enhancing existing coping mechanisms such as religious beliefs, support networks and physical exercises among YPLHIV can counter the physical and psychosocial effects of living with HIV and improve well-being. The importance of developing a healthy body image for YPLHIV should be emphasized in both health and family settings.

## Supporting information

S1 Appendix Data (interview transcriptions). (DOCX)

S2 Appendix Interview guide. (DOCX)
