# Supplementary material for: “I like the way I am, but I feel like I could get a little bit bigger”: Perceptions of body image among adolescents and youth living with HIV in Durban, South Africa
Source: PLoS One. 2020 Jan 10;15(1):e0227583. doi: 10.1371/journal.pone.0227583 (PMC6953798; doi:10.1371/journal.pone.0227583)
Supplement: S1 Appendix — (DOCX) [file pone.0227583.s001.docx]

**Interview 5**

Interviewer: Okay Innocent can you please tell me eh...your age.

Interviewee: I am 16

Interviewer: You are 16. Okay I am sorry, I am starting to record our interview. So, my first question is about feelings and thoughts on the appearance of your body. So, can you please tell me how would you describe the way your body looks?

Interviewee: Em, when I have just found out that I was HIV positive (eh), there was some symptoms in my body, I started developing some sores (eh) it wasn’t clear at first what was happening with me (eh), the first time I was told that I have TB (eh) I was treated for TB, then after some months, it was discovered that I’m HIV positive (mm). I was skinny, then I started taking medication (mm) and I saw my body recovering, the sores disappeared (mm), I started gaining weight (mm, okay).

Interviewer: So, when you say you were skinny, were you skinny naturally or it’s because you were sick?

Interviewee: It’s because I was sick.

Interviewer: Because you were sick?

Interviewee: Yes.

Interviewer: But naturally, your body is not skinny?

Interviewee: Yes.

Interviewer: Okay. So, what are your thoughts on how your body looks?

Interviewee: Right now?

Interviewer: Yes, currently.

Interviewee: You mean my thoughts?

Interviewer: Yes, on how your body looks

Interviewee: Eh…it is okay now, I like it (eh) it’s just the spots of the sores (eh), but I am trying to remove them.

Interviewer: Okay.

Interviewee: Yah.

Interviewer: What is causing the spots…oh you said you didn’t have them before?

Interviewee: Yes.

Interviewer: So, how are you removing them, if I may ask?

Interviewee: Oh, my sister bought me some cream that I apply to them (oh) and it helps me.

Interviewer: It helps you

Interviewee: Yes.

Interviewer: Okay. So, does the way other people perceive your body-body affects you-affects how you feel?

Interviewee: No

Interviewer: How? Can you explain to me maybe?

Interviewee: Like people who are teasing me?

Interviewer: Yes.

Interviewee: No, they don’t tease me, instead they are trying to tell me what can help me (eh) yah, especially my friends (mm) yah.

Interviewer: Okay, I don’t know if you have understood the question well, but it says does the way other people perceive your body, affects how you feel, how like can you explain?

Interviewee: Aw…I don’t understand the question.

Interviewer: You don’t understand the question, okay, it says does the way people recognize your body or perceive it affects the way you feel about it?

Interviewee: Yes.

Interviewer: It affects you? How, if you can explain.

Interviewee: Like if a person can look at me for a very long time and doesn’t tell me what is he looking (eh), I ended up thinking that maybe he is looking the way my skin is (eh) yah.

Interviewer: So, it makes you feel…

Interviewee: Uncomfortable.

Interviewer: Uncomfortable, oh okay. So, despite that then, are you satisfied with the way your body looks?

Interviewee: No.

Interviewer: No, why? (Laughter) can you explain why are you not satisfied?

Interviewee: Um, can you repeat the question?

Interviewer: I am asking if you are satisfied-are you satisfied with the way your body looks or the way your body is?

Interviewee: Yes…

Interviewer: Yes?

Interviewee: I am satisfied.

Interviewer: Can you please elaborate on that.

Interviewee: But, I am worried with the spots only (eh) I like the way my body is shaped

Interviewer: Wow! (Laughter)

Interviewee: Yah

Interviewer: So, it’s the spots only that you are worried about?

Interviewee: Yes.

Interviewer: Okay, I get you Innocent. So, we are going to the second par of the questions, whereby it focuses more on people who are significant in your life, on how…their perceptions of how your body…of how your body image influence your attachments with significant others. Like, okay its perceptions of how body image influences attachments with significant others, like the way your body is, how do you think it influences attachments with people who are significant in your life? So, my first questions says, does the way your family, friends, partner think about your body affects you in anyway?

Interviewee: No.

Interviewer: No. why? Maybe we can start with your family. Like, does the way your family think about your body affects you?

Interviewee: No

Interviewer: You are saying it doesn’t affect you? Why?

Interviewee: It doesn’t affect me because they don’t know what’s causing this in my body.

Interviewer: Oh, okay.

Interviewee: Yah.

Interviewer: So, what about friends?

Interviewee: No.

Interviewer: It doesn’t affect you, why do you feel that way?

Interviewee: I feel that way, because they are also saying that spots is something common, you can just develop something without know its cause (yah) then it will disappear and leave a spot (mm) Yah.

Interviewer: Can I ask, are you dating someone?

Interviewee: Yes.

Interviewer: So, does the way he think about your body affects you in anyway?

Interviewee: No.

Interviewer: No, why?

Interviewee: Because he tells me that “I don’t have a problem with the way you look, (wow!) I just love you” (mm).

Interviewer: That’s-that’s great! So do you think the way your body looks, affects the way others accept or reject you?

Interviewee: Can you explain it in IsiZulu?

Interviewer: Like, do you think the way your body looks, make other people to accept or reject you?

Interviewee: They accept me.

Interviewer: They accept you?

Interviewee: Yah.

Interviewer: Mmm

Interviewee: Because the body doesn’t say much about you (eh) you see (eh). The spots too says nothing, they just accept me the way good I am (eh), especially my friends and my family (mm) they love me a lot (mm) they say I have a good heart.

Interviewer: So, in other words you mean your body appearance doesn’t matter, but it’s your personality or your attitude that can make people accept you or reject you?

Interviewee: Um...it’s my attitude.

Interviewer: Your attitude. So, have you ever experience a situation whereby you have been rejected or you felt rejected because of your body, like since I am this size that is why people do not accept me or these kind of people are not accepting me or do not love me??

Interviewee: Can you please repeat the question for me?

Interviewer: I am asking if you have ever felt like you are rejected because of the way your body looks or how it is?

Interviewee: Yes.

Interviewer: You once felt like that

Interviewee: Yes.

Interviewer: How, can you please how it happened?

Interviewee: You mean whereby I undermined, like I undermined my body?

Interviewer: No, I am asking if you have ever felt like people do not like you or reject you because of how body looks. Like, for example, if you can feel like this person doesn’t like me, or doesn’t accept me because my body is fat, things like that you see.

Interviewee: No, I have never come across that situation.

Interviewer: You have never experience such situation.

Interviewee: Yah.

Interviewer: So, aw…oh….okay, what makes you don’t trust them, because I understand that you have just met your boyfriend, but friends are people you have been living with, you grew up together, so what makes you not trust them?

Interviewee: Em the problem is I have just met the friends I have because I have relocated from where I was staying before.

Interviewer: Oh okay

Interviewee: Yah.

Interviewer: So, what about the old ones, because I know you guys are still in touch with each other.

Interviewee: Yes, they know about this, because I am not the only who have it amongst them.

Interviewer: Okay, I am sorry if this will sound too personal, but how long have you been taking the treatment?

Interviewee: Em… since 2010

Interviewer: Since 2010?

Interviewee: Yes.

Interviewer: Oh. Okay alright. I get you sister. So, how does positive or negative feedback from significant others on your body appearance makes you feel?

Interviewee: Can you expain it in iSiZulu?

Interviewer: Okay, how does positive feedback (mm) or negative feedback from significant people n how your body looks makes you feel?

Interviewee: People who are too close to me?

Interviewer: Yes, close people, be it a boyfriend, family or friends, like how does their positive feedback or negative feedback about your body, on how your body looks or your body appearance makes you feel?

**Interview 6**

Interviewer: Uh okay Percy, thank you so much, we are about to start doing our interview, and you can feel free, you can also speak in IsiZulu its fine. So, before we start, can you please tell me your age?

Interviewee: I am 19 years old

Interviewer: 19 years?

Interviewee: Yes

Interviewer: Okay. So, okay…umm..umm….for our questions, I would like to ask you about the appearance of your body on how it looks and thoughts and feelings of young and adolescents people who are living with HIV. So, how would you describe the way your body looks?

Interviewee: I have a small body, I am light in complexion (yah) emm I do not care what people can say about my body, like saying I am thin and all those things (yah), because we are not the same (mm) some are fat and some are thing (mm), so if you say I am thin I will ask if you have ever seen me fat-fat (eh), so I don’t have a problem about my body, the problem is that I don’t get the kind of respect I deserve (eh) I end up being approached by kids and when I look at that little thing I wonder if he doesn’t realize that I am old (laughter, eh). So, since I have a child now (eh) I feel happy if someone calls me Melo’s mother (eh), I can see that person is respecting me (eh), so yah!

Interviewer: Oh, alright. So what are your thoughts with the way your body looks?

Interviewee: Umm…I like the way I created (mm) yah I like the way I am, but I feel like I could get a little bit bigger instead of getting too small you see (eh) just to gain a little bit in my body, so I used to get sick, lose weight and gain it again, so that how it is.

Interviewer: Oh, okay, what makes you want to get a little bit bigger?

Interviewee: (Silence) what can I say, eish…if you look too small, ey…

Interviewer: What’s wrong with it?

Interviewee: Eish, no, especially when you are wearing a dress and if it’s too tight on you, you can also that it doesn’t look fine (eh), and when you look at yourself in the mirror you can see that no, I am looking too small now (yah), I don’t know how I can explain this thing (eh)

Interviewer: Okay, so you do not feel right?

Interviewee: Yes, I do not feel alright when I’m too small (yah).

Interviewer: You have mentioned earlier that at times you get sick and lose weight, what is the cause of that?

Interviewee: Maybe if I am facing some challenges like-if…for example at home, if I have some issues with someone at home (yah) because it common that families have some disagreements (yah) and then solve that issues, so I have that issue that if I am going through hard times, I lose weight (okay), if there is something that is bothering me, I lose weight and pick up again when things are okay (okay, yah it happens) mm.

Interviewer: So, do you think the way people perceive your body or they look at your body affects the way you feel about your body?

Interviewee: Mmm…sometimes

Interviewer: Why?

Interviewee: Because I also end up undermining myself, and when I look at myself I feel like I have lost too much weight, especially if I have been sick and lost weight (eh) that is where I undermine myself and feel like I do not like myself, except when…you see right now, I can feel myself (mm) yah.

Interviewer: What does undermining yourself means?

Interviewee: Undermining yourself means you end up doubting yourself (oh) lack self-love (okay, okay).

Interviewer: So…alright. So, if-if-if you find that people are not treating you well based on the way your body looks you don’t feel happy about it?

Interviewee: Yes

Interviewer: So, are you satisfied with the way your body looks?

Interviewee: Yes, I am satisfied.

Interviewer: You are satisfied. Why are you satisfied?

Interviewee: (Silence) what can I say…

Interviewer: Or let me say what makes you feel satisfied with the way your body looks?

Interviewee: When I am dressed, I see myself as beautiful (eh), so I even feel that the outfits that I am wearing are looking good on me (mm) in this kind of body I have (yah, I get you).

Interviewer: Okay, earlier you have mentioned that people are saying you are thin and make all those funny comments about your body and that other people’s perceptions towards your body makes you doubt yourself, and then now you are mentioning that you can feel yourself when you are dressed up. So, I do not if I am getting you well here, like when do you exactly feel like-feel like your body is not okay, like maybe is it when you’re well-dressed or not?

Interviewee: You see when I have been sick and lost weight (yah) that is where I start lacking self-love (eh), then if I have picked up and gained some weight, let me say maybe I have lost weight and end up wearing size 28, since I am size 30 now (mm) that is where I lack self-love (mm). If a person says I am thin, I always remember that indeed I have lost weight (alright, I get you)

Interviewer: Ah, alright, so the next questions will be about significant people in your life based on their perceptions-perceptions about your body, the way your body looks and how do they influence-influence your attachments with them. So, like does the way your family, friends, partner think about your body affects you in anyway? The way maybe your family, friends and your romantic partner thinks about your body, does it affects you?

Interviewee: People that I am closed to have never said any nasty comments about my body, it’s only those that I have no attachments or relationship with them, that they keep saying bad things about you (eh) the ones that I have a relationship with do not have any problems with me (mm) even my family. If for instance I have lost weight and I tell them that I feel like I have lost weight, they try to take that out of mind and say “no, you haven’t lost weight (eh) you are not thin, you are okay (mm) yah.

Interviewer: I would like to ask if you have any romantic partner.

Interviewee: Yes I do.

Interviewer: Eh..umm…what is he saying about your body?

Interviewee: He doesn’t have any problem with my body (eh), but…what can I say, he also tells me perhaps if I have lost weight (eh) he say “you are losing weight, you must try doing this and this” (eh) Yah (okay)

Interviewer: Eh…I would like to ask if he knows about your life status.

Interviewee: Yes he knows about it.

Interviewer: He knows about your life status

Interviewee: Yes.

Interviewer: And how is his status?

Interviewee: He went for testing and they said he is fine.

Interviewer: Oh, okay alright. So, how did you disclose your health status to your partner and when? Like can you please explain to me about that thing?

Interviewee: Okay. It was, it was hard though (eh) because you won’t find it easier to trust a person, so we have been in love for a very long time before I disclosed to him

Interviewer: Like for how long?

Interviewee: Maybe 2 months.

Interviewer: Okay.

Interviewee: When did we start dating though? Oh, we started dating on September, so it was September, October and November, and then I told him in November. Oh no I’m lying, I told him in Dcemeber, that I was born with it. It was not easy to…because I am that person who finds it difficult to talk (eh) so if there is something that is bothering me, I feel like writing it down (yah). That is why I have a diary that I keep writing about my daily lives, like today this and that has happened like this and that (yah). So, I told him that there is something that I always wanted to tell you, but when I’m with you things get sweet and I get reluctant to tell you because I thought it would spoil our happiness (mm) and then he said “tell me because if you don’t tell me we will end up fighting” (mm). So, I asked him to give me his phone, and he gave men the phone. Then I told him that I can’t say it with my mouth because I am scared, so I will write it down. Okay, I wrote it down and I wrote everything, even though I don’t remember what I wrote, but I also told him that I will accept it if you leave me things like that (mm). And then he said “I will never do that thing, because there are lot of people who are living with this virus (yah, yah) you see, if I leave you, I will find someone who will not disclose it to me” (mm), he said so. He also said “what I like-prefer-what I like is that you were born with it, it is not…what can I say, you didn’t ask for it (yah) it’s not like you asked to be like this (yah), you have never went out and look for it outside (mm), you were born with it, I will not change that and I accept you (mm). Then we continued dating, I got pregnant and gave birth in 2017, we started dating 2016 (ow).

Interviewer: I get you sister. So, do you think the way people look at your body makes you feel accepted or rejected? Or do you think that the way your body looks make people to accept you or reject you, because of your body?

Interviewee: Ey, for some, they accept me and some do not

Interviewer: Why is it so?

Interviewee: They are kind of talking that I am sick and things like that you see (mm), especially those who knows my family (mm) are the ones who talk a lot about me that I am thin because I am sick (mm).

Interviewer: What do you mean when you say those who knows your family?

Interviewee: I mean those people who were too close with my mom and dad (okay), especially my dad, because I’ve been told that he used to talk a lot about his status (ow) he wasn’t keeping it to himself (okay)

Interviewer: So it’s those people who makes you feel they are rejecting you

Interviewee: Yes.

Interviewer: Do you feel that people are accepting you because of the way your body looks?

Interviewee: No.

Interviewer: Okay. So, how does positive or negative feedback from significant people in your life on how your body looks makes you feel?

Interviewee: (Silence)

Interviewer: Like when they are giving you positive feedback, like complementing your body, how does that makes you feel, especially if it comes from significant people in your life?

Interviewee: It makes me feel happy.

Interviewer: Why is it making you feel happy?

Interviewee: (Laughter) it is nice to be complimented at all times (yah) yah it is nice.

Interviewer: Yah, it is nice. So, okay if they are giving you negative feedback or something that is not nice about your body, how does that makes you feel?

Interviewee: Ey…I don’t know.

Interviewer: You don’t know? (Laughter)

Interviewee: I have never been in that position.

Interviewer: You have never been in that position?

Interviewee: Yes.

Interviewer: Okay. Okay so if I can say, maybe you feel like someone is saying bad things about your body, how does that makes you feel then? Or if you realize that a person is looking at your body in a bad way, how does that makes you feel?

Interviewee: Ey, it doesn’t make me feel good, because there was a time where I have relocating from the G-section (mmm) then I went to visit somewhere, okay, and some guy said to me…he even knows that I have a baby daddy (mm), and he did something strange (mm) and he wanted to do…you see

Interviewer: Something strange, as in how?

Interviewee: Touching my private parts.

Interviewer: Okay

Interviewee: Yah. Then I asked myself why, do I look cheap on people and that thing bothered me a lot (yah).

Interviewer: Did you manage to tell that guy how he made you feel?

Interviewee: I-I didn’t tell him, I told my partner what happened (oh, okay alright). They then called him and sat him down and he said him too doesn’t know what happened (mm).

Interviewer: Maybe he was attracted to you.

Interviewee: I don’t know, he made me feel like I was cheap (yah, yah).

Interviewer: What made you feel you were cheap, because guys have a tendency of touching girls anyhow?

Interviewee: Hell no! A person can’t just touch you anyhow knowing that you are staying at your baby daddy’s house, that you are in love with (yah) you see that thing (okay), I don’t know how to explain it.

Interviewer: So, you are staying at your baby daddy’s family?

Interviewee: I had been visiting there.

Interviewer: Oh okay, by that time?

Interviewee: Yes, by that time.

Interviewer: oh, okay

Interviewee: So he came there, and he was used to visit there because he was a friend with one of them whom they were studying together, as they would be doing schoolwork, writing their homework, you see all those things (yah). Then one day, where no one was at home, so they asked him to come keep me a company, because it was late in the evening and he would go back to his house once they were back (yah), that is where he started touching me (okay).

Interviewer: Can I please go back a little bit, about you and your baby daddy (mm), you said, you said now um… you are not using a protection (yah) since…since you started that night (yah), so even now you are not using a protection?

Interviewee: Yes.

Interviewer: No, why are you saying so?

Interviewee: (Silence) (laughter) I-I…what can I say, I can make friends easily, despite how my body looks, whether thin or how (yah). I have all kinds of friends (yah) whether they have big bodies or how (yah) I don’t care about that.

Interviewer: So, there isn’t a time where you feel like you can’t hang up with these kind of people because…

Interviewee: It has fat people?

Interviewer: Yes.

Interviewee: No (laughter), all my friends are fat, I am the only one who is thin (laughter) (okay)

Interviewer: Since you have said that it is easier for you to make friends, don’t you encounter some difficulties of maintaining that relationship you have built?

Interviewee: No, it doesn’t.

Interviewer: It doesn’t happen

Interviewee: Yes.

Interviewer: Everything gets fine, you don’t feel like you are being discriminated or something like that?

Interviewee: No, it doesn’t happen.

Interviewer: Okay. You are always feeling welcomed?

Interviewee: Yes.

Interviewer: Okay

Interviewee: Because when I was…since I already had a child when I was doing Grade 12 (mm), so I met this girl who got a child earlier than me (mm), and then she asked to speak with me privately, and she told me that she had this problem (mm) and she said “I am HIV positive and I have never told my baby daddy, I am scared to tell him” and I said to her, wow! We are on the same situation and I realized that there is at least someone who is like me, who is also HIV (mm) so I advised her to do one and two (mm).

Interviewer: What did you say to advise her?

Interviewee: I told her how I disclosed to my partner (yah) and then I said to her it doesn’t help hiding it from him, especially now that there is a child. So I advised her to sit his partner down, tell him and even mention that I too also have HIV because he knows me (mm) mm

Interviewer: What do you think is the importance of disclosing your status to your partner?

Interviewee: Pardon?

Interviewer: Why is it important to disclose your status to your partner, why can’t you just keep it to yourself, why are you supposed to disclose your status to your partner?

Interviewee: It won’t be a good thing if he finds out by himself (mm), because he has to take an informed decision whether he continues dating you or not since you are HIV positive, instead of you forcing him, because he doesn’t know, if you haven’t told him he doesn’t know that you are sick (ow), so that he can also get some treatment if he has been already infected (okay, I get you, I get you)

Interviewer: Do you think the way your body….sorry, do you think significant people in your life influence the way you are satisfied with your body?

Interviewee: Yes

Interviewer: Yes? Why do you feel they are influential?

Interviewee: I don’t know what to say here….. (Silence)

Interviewer: Pardon?

Interviewee: Let’s move to another question (laughter). I will come back to this one

Interviewer: (Laughter) okay we will come back it is fine. Then we are now going to talk about resilience, like how do you manage to deal with the challenges that you are facing?

Interviewee: Pardon?

Interviewer: Like, how do you manage to cope with the challenges (ow) like all the bad things that people are saying about you or your body? So, how do you manage to accept your body as it is? How do you accept your body the way it is and everything about it or yourself?

Interviewee: It is difficult.

Interviewer: It is difficult, why are you saying it is hard or what is it that is difficult?

Interviewee: Especially…firstly, it is hard to accept that I am HIV positive (mm), I always ask myself why me? (Eh) and ask why my parents was-was…I don’t know what to say (mm). Why did my parents kept that from me, because my parents are dead, why they didn’t tell me, until I find out myself. I am staying with my grandfather now (mm) I have been sick and sick until I find out about this thing, because they knew about it, they knew.

Interviewer: So you got sick when you were living with your parents or with your grandfather?

Interviewee: When I was staying with my grandfather, after they died.

Interviewer: Can I ask that…when you find….when did you find out, did you just find out or?

Interviewee: Mmm…I find out when I was 7 years.

Interviewer: Oh, okay

Interviewee: So, I am saying why they didn’t tell me before they died because they knew (mm) that they are HIV positive (mm).

Interviewer: So, you have been sick several times, and then in and out of hospitals until doctors recommended that you do HIV testing or you just did the testing by yourself?

Interviewee: Okay, my grandfather said…no no no, it’s this thing that they do in schools, whereby they will come to do testing (mm), then I tested and I discovered that I am HIV positive, then they said I must go to a clinic and they recommended the Blue Roof clinic (mm). Then I went to Blue Roof for testing, and that is where I started taking my treatment.

Interviewer: You started from that time

Interviewee: Yes.

Interviewer: Oh, okay. How did you know that your parents knew about your status?

Interviewee: I used to hear it when the family is talking because I heard that my father used to talk a lot about his status, so I assume he knew. Then, my mother I know that she died from HIV, I cannot blame my mother that much, but my father you (yah).

Interviewer: And they didn’t tell you?

Interviewee: Yes.

Interviewer: So if people are making hurtful remarks on how your body looks or about your body, how do you manage to deal with that situation?

Interviewee: Pardon?

Interviewer: If people are making hurtful remarks about your body, for example, like saying you are dark skinned and fat, like bitter things about your body (mm) how do you deal with those things or how do you manage to cope with it?

Interviewee: I am that kind of person who will just cry if something is bothering me and then I will let it go.

Interviewer: You cry and let it go, so you do not have anyone that you can talk to?

Interviewee: There is, but at times there things that I do not feel comfortable to share them with other people. For example, telling someone that I have met a person who made nasty comments about my body, staff like that (mm), no I don’t share those things, I will share about serious things that I need an advice not such silly things.

Interviewer: But at times you might be just going in the road and then meet someone who will say bad things about you and you won’t get that space to cry, because you need privacy to cry (mm), so how do you deal with such situations, for example if you can meet a person at Shoprite and say you are thin, how do you deal with that situation at that times, for instance you don’t have privacy for you to cry, because you can’t go crying in public.

Interviewee: I keep quiet.

Interviewer: You keep quiet?

Interviewee: I don’t respond.

Interviewer: Okay, so besides crying, what else do you do when something is bothering you? You have mentioned that you jot it down in your diary (yah), and you cry, so there is no other thing that you do like…

Interviewee: There is none.

Interviewer: There is none, okay. Okay Percy, so do you have any other people in life who listen to you when you are talking, who gives you support when you need it?

Interviewee: Yes they are.

Interviewer: Which are those people?

Interviewee: My grandfather (eh), my grandmother (eh) and my baby daddy.

Interviewer: Okay, why is it them?

Interviewee: I don’t know, maybe it’s because e they understand my life, they know well that I am like this (mm)

Interviewer: So, you feel free when talking to them, you feel they are not judging you?

Interviewee: Yes.

Interviewer: What about friends?

Interviewee: Friends…I haven’t told them, it’s only this one because we have the same problem

Interviewer: That you were able to disclose to

Interviewee: Yes.

Interviewer: Why are you not telling your friends?

Interviewee: I am scared

Interviewer: (Laughter) why are you scared?

Interviewee: They will judge me.

Interviewer: They will judge you?

Interviewee: Yes.

Interviewer: So, you can’t talk to them?

Interviewee: Yes, I can’t.

Interviewer: Okay. Thank you so much Percy, a lot. All the information you have given us is very important and it will be so helpful. Em…I would like to ask…can you please ask me if you have any question for me, or if there is something you may like to add on, or if there is something you may like to suggest.

Interviewee: Ay, there is none.

Interviewer: There is none?

Interviewee: There is none.

Interviewer: You do not have any question for me?

Interviewee: No, I don’t have

Interviewer: Okay. Can we please go back to that question (laughter) that we have skipped? So, do you think significant people in your life are influencing you to be satisfied about your body? Do they have an impact on how satisfied or happy you are with your body?

Interviewee: My problem is I do not know how to answer this question.

Interviewer: I don’t know maybe I am not explaining it well.

Interviewee: Maybe you are explaining it well.

Interviewer: Okay, this question is talking about significant people or who are very important in your life (yah), so this question is asking if they influential that you are happy or you are satisfied with your body? Like if you can say you are happy with your body, is it because…

Interviewee: Because of them?

Interviewer: Yes.

Interviewee: (Silence) (laughter) Yah!

Interviewer: Yes, why yes? Can you please explain a little bit?

Interviewee: Their advises that I receive from them, perhaps if I have met someone who said hurtful remarks about my body, and when they are telling me that I am beautiful and staff like that (mm) I end up seeing myself that I am indeed beautiful and there is no need for me to change (yah, okay) yah.

Interviewer: Can I ask you the last question, okay beside people then, because you are mentioning that people supports you at time, so do you have that self-esteem and self-love towards your body?

Interviewee: I do.

Interviewer: You have that thing? Okay. Because it is very important and I hope you understand why it is important.

Interviewee: Yes.

Interviewer: Thank you so much Percy, we are done, but I would like to ask again if you have any questions, just in case you have thought about asking me any questions or anything you may like to add.

Interviewee: No, there is none.

Interviewer: There is none. Okay thank you so much sister.

**Interview 8**

Interviewer: So, basically eh for the first question like I would want to hear like okay when you…you look at yourself or your body how would you describe the way it looks.

Interviewee: I think um…

Interviewer: By the way there are no wrong answers, it’s just what you think.

Interviewee: I would say it looks normal

Interviewer: Okay

Interviewee: But at some point, knowing your status you always feel that like you are different (mm)

Interviewer: At some point?

Interviewee: Yes.

Interviewer: So, maybe in terms of feeling different, is it something maybe that is physical that you can see or is something that you think about?

Interviewee: Mostly it’s something that you think about it

Interviewer: It’s something that you think about?

Interviewee: Yes

Interviewer: Okay, so, in that case then uh does that really impact or does it have a side impact on how you feel about your body. The fact that maybe you just think about it, does it have an impact on how you feel when it comes to that?

Interviewee: Yes, it does (mm) yes it does because at some point, some other things you can’t do it (mm) you know, yah.

Interviewer: Like?

Interviewee: Enjoying life without a….(laughter)…. (Okay)

Interviewer: So, maybe do you sometimes feel maybe like okay it limits you from maybe doing certain activities maybe like you are saying that maybe your friends are saying lets go

Interviewee: Yah, yah I would say it limits me

Interviewer: It limits you? So, maybe if you find yourself like in that position, how do you deal with it?

Interviewee: Making up some excuses (laughter) or not going (okay) and then I will stay inside my house.

Interviewer: So, you come up with an excuse?

Interviewee: Yes, I come up with an excuse (okay)

Interviewer: Okay. Yah I’m sure it works most of the times

Interviewee: Yes, it does.

Interviewer: So, okay like um going back like the to the question like do you feel maybe that the way that other people perceive you or look at you, does it really have an impact on how you feel about yourself?

Interviewee: No.

Interviewer: It does not?

Interviewee: I do not think so.

Interviewer: You do not think so?

Interviewee: Yes.

Interviewer: So, maybe after like, in general, would you say like you feel satisfied about your body?

Interviewee: Yes, I am.

Interviewer: You do feel satisfied?

Interviewee: Yes.

Interviewer: So, like even like in those times maybe when you get to think or be the fact that maybe you might be living with HIV, does it affect how satisfied you are?

Interviewee: It does, but you know

Interviewer: Not that much?

Interviewee: Yes, not that much because it is something that I have already accepted.

Interviewer: Accepted?

Interviewee: Yes.

Interviewer: Oka, okay. Okay that is good. So, moving on to the next eh part which I find to be quite interesting (okay). So, I think you have said you have a partner.

Interviewee: Yes, I do.

Interviewer: Okay, you have been dating for how long?

Interviewee: It’s been two years now.

Interviewer: 2 years?

Interviewee: Yes

Interviewer: And like uh, how was it like, have you been opened to disclose it to her?

Interviewee: No.

Interviewer: You haven’t?

Interviewee: No, I haven’t.

Interviewer: And how does that make you feel?

Interviewee: It…makes me feel like I am some kind of a boring person

Interviewer: Bullying?

Interviewee: Boring, because you know how girls are like, they like to talk about their relationships and the staff (mm) with other girls, so when it comes to things for example sexual activities, maybe they are talking with their friends and are being asked (mm) if they have been involved with a man, so ya, at some point at this stage I am not going for it.

Interviewer: Okay, so like you do not talk about things like sex?

Interviewee: I do (mm) but when it comes to doing it (mm), it’s another story.

Interviewer: It’s another story?

Interviewee: Yes.

Interviewer: Yah, so like um I am sure maybe as you are dating this person, maybe you have intentions of making this person your girlfriend or your wife at some point, or maybe let me put it like we know as boys that there is a time where it gets like okay I feel like to have sex with this person.

Interviewee: Yes, I do reach that point, eh but as I said earlier (mm) you know I always like create something (excuse) that we do not have sex.

Interviewer: So like uh…for how long do you think you are going to…

Interviewee: Playing this game?

Interviewer: Yes.

Interviewee: As much as I can play it, I could say. The fact is I cannot say it right now (mm) because we are still young (okay, okay), so if you like open and disclose about my status to this person at this age you know

Interviewer: It is too early?

Interviewee: It’s too early yah (okay), you wouldn’t know what that person will do.

Interviewer: Will do, on how they will react?

Interviewee: Yes, so I think maybe in later stages (okay).

Interviewer: So, you have said you have been dating for like 2 years, so you are saying maybe when you are getting maybe to the third or fourth year you will be able to tell her.

Interviewee: I think it’s too early.

Interviewer: Too early? (Laughter) how log, how long then do you think?

Interviewee: Um…I think when I am financially stable, yah (okay). I do not want to add another crisis when we are already having our own right now.

Interviewer: Which crisis do you have now?

Interviewee: You know, practical staff (laughter) taking her out, going out (oh yah, yah), yah having sex and all that, so yes, it’s those general staff.

Interviewer: But of course, you go out isn’t it?

Interviewee: Yes, I do take her but….eish (laughter)….

Interviewer: For yourself

Interviewee: Yes.

Interviewer: So, are you going to school or?

Interviewee: Yes, I am going to school.

Interviewer: You are going to school?

Interviewee: Yes.

Interviewer: Okay, I understand. So, you said of course that you haven’t like, so when you started dating your girlfriend was it or after you already knew, or it was before?

Interviewee: it was already.

Interviewer: Already?

Interviewee: Yes.

Interviewer: So, would you say maybe in that point like since you already knew, like did you think about it when you are-when youwere trying to ask this girl, like did you feel like okay would this…I am trying to say did it limit you or did you have that case that okay but I am living with HIV, so should I ask this girl like?

Interviewee: No (mm), it doesn’t. at that point when you meet someone it doesn’t.

Interviewer: It doesn’t?

Interviewee: Yes.

Interviewer: You just feel like it’s fine I can

Interviewee: Yes, because you only for that…(for the…) (Laughter)

Interviewer: That…

Interviewee: Yes.

Interviewer: It doesn’t?

Interviewee: Yes.

Interviewer: I get it. And um…with your current relationship, what do you think is or what can you say maybe is the most difficult part?

Interviewee: Being, having sexual activities (mm) I think is the most because of her friends (mm) are the biggest influence, (they are all) they are having sexual activities (mm), so she is like the only one that is left

Interviewer: So, she wants to do it as well?

Interviewee: Yes, she wants to do it.

Interviewer: And um because I mean like with sexual activities covers a lot of things and people they do kiss and do touch which is not really sexual activities (yes) but do you get to do that at least?

Interviewee: Yes, I do.

Interviewer: Okay. And so I know you have said like you can come up with excuses for not wanting to do it, but what are some of the excuses, like let us say maybe you are chilling, and if you get to kiss and you get to touch, how do you then stop it from getting into that?

Interviewee: To that point?

Interviewer: Yes.

Interviewee: Um…usually I would say that I am not ready (mm), and at some point, we should get married (laughter) and I make it at a point that she understands it that I am having this situation that I need to deal with it right now (okay), so like coming up with excuses or something (okay, okay) yah.

Interviewer: And like of course, I know that you have said that it does work for you, but don’t you feel scared at some point that maybe she will get fed-up with you and then

Interviewee: I do at some point because she has tried many times (mm) so yah.

Interviewer: So, does that bothers you?

Interviewee: It does, but you know it’s life, you can’t really accept this thing.

Interviewer: So, what if you lose her like..?

Interviewee: It will affect me (mm), but not that much.

Interviewer: Not that much?

Interviewee: Yes.

Interviewer: You would be prepared to go (yah) for someone else?

Interviewee: Yes (laughter), I do not think it will affect me that much (mm)

Interviewer: Okay, so, like when you meet with your friends, even in your side when you are with your friend, even on your side you know like as you have said that everyone has done it and I am sure your friends have asked you like have you done it or?

Interviewee: Yes, they do ask about it sometimes (laughter).

Interviewer: And how do you respond?

Interviewee: At some point I lie (mm).

Interviewer: That you have done it?

Interviewee: Yes, to avoid any complications.

Interviewer: Complications?

Interviewee: Yes, at some point I do that, but only one of my friends I tell him the truth.

Interviewer: Okay. Why that one, like is he a close, close friend or you know that he won’t judge you?

Interviewee: Yes, because he is like my brother to me.

Interviewer: He is like a brother to you?

Interviewee: Yes, like and he is even more than a best friend.

Interviewer: Okay, you are very close with that person?

Interviewee: Yes, and I know she doesn’t, he doesn’t judge a lot (okay) yah.

Interviewer: So, like uh with that person who is your friend and the one you are calling more than a brother, does he also know your status?

Interviewee: Yes, he does.

Interviewer: Oh he does?

Interviewee: Yes.

Interviewer: And like I am sure he has been so close to you, like what kind of an advice does he gives you especially when it comes to that situation of between you and your girlfriend?

Interviewer: Support you with this?

Interviewee: Yes.

Interviewer: Oka, okay. I do understand, and I am glad that you have that one person at least you know that this person has you back and you know that you can talk with them anytime you feel like doing so. And I know I have already asked this question and you have said that you are satisfied with your body, so there is no way like you feel maybe at some point you were feeling like a bit like too sick?

Interviewee: Um…it’s when like eventually…um…when I had to go and do test (mm), in that year I was already sick.

Interviewer: Already sick?

Interviewee: Yes.

Interviewer: So like um… during that period when you were sick, did it affect your body?

Interviewee: Yes, I did and my weight

Interviewer: You lose weight?

Interviewee: Yes, I lose weight and I coughed a lot, even coughed blood and I thought I had TB at that time (okay), yes, I couldn’t do anything (okay).

Interviewer: But the, since then up to this period do you feel like things have changed with your body?

Interviewee: I think yes it has changed, but it’s something I do not put on my mind (okay) so...

Interviewer: So, in other words you are saying, even though you know that um something is changing, you do not really try to focus on it, but you try to ignore it the best?

Interviewee: I try to ignore, but you when you like facing like a challenge (mm), so it always like popping up again.

Interviewer: Okay, so, how do you deal with it like the fact that it is always popping up, but how do you deal with that?

Interviewee: Sometimes I will walk (mm)to the river because there is some river (mm) across to get myself in the… that you know what I still have to live because there is a purpose I am here for (mm), so yes, that is the only think that I do.

Interviewer: And that keeps you going?

Interviewee: Yes.

Interviewer: Okay

Interviewee: Because if I take a look at things, even my background is not good (mm).

Interviewer: So, maybe, maybe of course when you are going through these difficult times, do you maybe take time to share with your friend, the one you said he is almost like a brother?

Interviewee: Not all of them.

Interviewer: Not all of them? So, how do you deal with other staff that you do not tell him, how do you deal with it?

Interviewee: I always try to find something to do at that point.

Interviewer: So, you try to do something, like to destruct yourself?

Interviewee: Yes, something more like that (okay), so I always try to do something like reading a book (mm) and doing some activities that will make me forget.

Interviewer: Forget those things?

Interviewee: Yes (okay).

Interviewer: So, probably maybe let’s say it was a different situation or maybe you were given an option, like what would you think would be the best way or what would you want to happen to you maybe to feel good or for you to deal with these things that you think about?

Interviewee: If I was given an option?

Interviewer: Yes. For example, as you have said that your family doesn’t understand, maybe you can say if only my family understood, then I would be open to tell them.

Interviewee: Yes, that’s the one, but at some point, I still think that you know the mistakes that I have made (mm) I think I regret it at some point (okay) yah

Interviewer: So, in other words you are saying maybe if you could go back you would want to change what has happened in the past?

Interviewee: Yes (okay)

Interviewer: Okay, I understand that but maybe looking at what is there like currently, is there anything maybe that you would want to happen or something that you would wish if it was like this then it was going to be easy for me?

Interviewee: Currently?

Interviewer: Yes.

Interviewee: No.

Interviewer: So, you are saying maybe everything, for now you are managing, you are coping well?

Interviewee: Yes, I am coping well.

Interviewer: You are coping well?

Interviewee: Yes.

Interviewer: Okay in that case then it makes sense then. Okay and the other question that I want to ask, but you have somehow touched on this, like do you have that person or people in your life that you feel like okay whenever I am going through something, this person is there to support me, and this person is there to listen to what I have to say?

Interviewee: Yes, I do, because if I want to talk (mm), I would say the community that I live in they judge a lot.

Interviewer: A lot?

Interviewee: Yes, so due to my background (mm), they do judge a lot (mm), but at some point, I feel like killing them (laughter), yes like I always reach that point like you know (mm), probably its life (yah, yah) so I would say it helps me a lot and makes things a better way to understand it.

Interviewer: Understand it?

Interviewee: Yes (okay), and at some point, they would encourage me that whatever that is happening because I think it’s in God’s plans (yah, yah, yah) yah.

Interviewer: And like um I’m sure what you have said that it’s God’s plan, you are kind of a religious person you believe.

Interviewee: No, I am not (laughter)

Interviewer: You are not really religious?

Interviewee: No.

Interviewer: But then…

Interviewee: Because is like I would be lying if I say I am because at some point when I am alone I do think why did God you know

Interviewer: Let this happen?

Interviewee: Yes (yah, yah, ayh), I have got so many problems that I have come across (mm) and he doesn’t like showing any help.

Interviewer: Any help?

Interviewee: Yes, so I do think that.

Interviewer: And ask yourself why, where is he in all these.

Interviewee: Yes.

Interviewer: Why is he letting all these happen?

Interviewee: Yes.

Interviewer: Yes, it makes sense. It makes sense that you have to ask God these questions.

Interviewee: Yes, it does.

Interviewer: So maybe in closing, I would be like what do you feel that would your views or what kind of your plans firstly like um with the situation that you have with your girlfriend like how do you see yourself maybe

Interviewee: In the next five years?

Interviewer: Yes.

Interviewee: Yo! Seems like I would say having a girlfriend is not a goal or something (mm), but you do need to have due to influence (mm), so I think when that times comes (mm), I want it to come when she’s already much matured (okay) to understand.

Interviewer: To understand?

Interviewee: Yes

Interviewer: So, basically you are saying you would want someone to understand your position and where you are?

Interviewee: yes.

Interviewer: And probably as you grow up, like people have like hopes and plans, I am sure you also have that hope that someday you would want to have your own family

Interviewee: Yes, I do (laughter) but sometimes when I research about it seems so difficult, I feel like, it’s like it wouldn’t really fair with me

Interviewer: Why not?

Interviewee: You know having kids living with it?

Interviewer: No, but I am not sure how much research you have done, but I know that even if two people are living with HIV they can have an HIV free child.

Interviewee: Yes, I do know that, but (mm) at some point, I think that is a miracle.

Interviewer: Yes, it might seem like a miracle, but I have been doing research a lot on this and there is something that is in fact most people, like 90% of most people are actually healthy children (mm), and some people who have a partner who is living with HIV and the partner who is not HIV positive, they can actually have a normal life as long as they use protection (mm). Unless it can be like a bit difficult having a life whereby you have to use protection all the time you have sex.

Interviewee: Yes.

Interviewer: It seems like a hustle?

Interviewee: Yes, …. (Laughter) it seems like a hassle for me (mm).

Interviewer: Why? Because like do you maybe feel like there is a difference when you have sex with a condom and when you have it without?

Interviewer: I think one question to ask is why should we always use a condom?

Interviewer: So, is it that girls usually like sex without a condom?

Interviewee: Yes, they do ask, why are we using it.

Interviewer: A condom?

Interviewee: Yes, because if they see you taking out a condom (mm) they will be suspicious…. (Suspicious, okay)…..

Interviewer: So like um maybe do you think that it would be easy where or way much easier, or actually it is difficult then telling someone, because I am trying to see which is much better telling someone that okay this is what is happening and then if you understand then it is fine, we can use a condom, or not telling that person and then you are always having to face this question whereby every time she is asking why are using, because I think maybe at some point you will tempted to say it’s fine and then not use it.

Interviewee: I don’t think it will come to that stage.

Interviewer: To that point?

Interviewee: Because I am trying to avoid where maybe she will end up being positive as well.

Interviewer: As well.

Interviewee: And then I would be responsible for that.

Interviewer: So but um don’t you think that maybe that the, the easiest way for her not to be positive as well is maybe to tell her at some point?

Interviewee: As I did say earlier (mm), at my age or at this stage

Interviewer: It feels like it’s too early?

Interviewee: Yes.

Interviewer: Okay.

Interviewee: Yah, because lot of them are still childish.

Interviewer: They are still young, not matured.

Interviewee: Yes, they talk a lot (yah, yah).

Interviewer: And obviously, I am sure you wouldn’t want to tell her that and then she will break up with you. You do not know if she is going to tell (yah) other people as well.

Interviewee: Yes.

Interviewer: Okay, okay it makes sense. I think I have covered like almost everything. Do you have any questions for me?

Interviewee: Mmmm

Interviewer: Feel free to ask.

Interviewee: Okay, can I ask why are you doing this research, because at some point you are doing, I think these questions are like general staff, like we always say a lot about them (about them), but you are still going out and doing research…..that we are having So, I am trying to figure out why?

Interviewer: Why?

Interviewee: Yes.

Interviewer: I think I would answer you, and like as you have said that you hear this a lot, like these questions they have been asked a lot, right?

Interviewee: Yes.

Interviewer: I would give you an example, like um you find that many people they have been told that uh they have to use condoms when having sex, but as you were saying that you go out there in the community and you want to have a girlfriend and you want to protect yourself at her, not that maybe you are HIV positive, but you just want maybe to do the right thing. But then the girl asks you why are you using this you see. So, the thing is that there is a lot of information out there, but people are not actually doing it, so with this research I am trying to okay see what is it that people out there know that okay, especially with the first question that I have asked that if there are people who are supporting you, we know that previously

Interviewee: They didn’t have support, they criticize a lot.

Interviewer: Yes, and like the evidence has been shown that if people are given that support, especially people who they live with, for example let us say your family knows right, and they will be like it’s time to take your medication, and you see maybe there. You want to say something?

Interviewee: Yes, I do think support it’s a good thing (mm, mm), but at some point it could be it’s a set where it reminds you where are you coming from (mm). you know it reminds you a lot having support, because at some people having support would be good, they will support you in a way that it will draw back things to make it pop up again. So, I think support is good, but not always.

Interviewer: Okay, can you give me an example of when like it might not be god.

Interviewee: You know I have visited a place, I think it’s last year (mm) and I came across where a 9 year old boy you know, I think he has defaulted like two times (mm), so when I came there to give that motivational advice (yes), and at that point I had to go to his family to see how are they treating him and staff (mm), and when I get there I finally saw why he did not take his medication (mm) because they are always saying he is going to die, go and he had to take your medication (oh, okay) and that was a little boy to understand that (to understand that, oh okay), so at some point it is discouraging too much (okay, okay).

Interviewer: Yes, yes I understand what you are saying and it does make sense if you are putting it that way, because I think maybe for me that might seem like a support, but it is not actually a support, because when people are supporting you, they are not supposed to use negative words, like you are going to die if you don’t (yah), because that might be the case, but they have to encourage you like saying “you know it’s time to take your medication, you have to eat healthy, you shouldn’t be smoking or you shouldn’t be drinking because it is not good for your health”. It should be done in a positive way (positive way) you see

Interviewee: But lot of them are not dedicated.

Interviewer: Yes, yes that is the thing and that goes back then to my...why am I doing research, because these are the things I would like to identify, like is it there a support, if it is not there, why is not there, if it’s there what kind of support are you being given, so that we can at least educate e or inform people so that they know why this is important. We also know that the is reason I am doing this research, you know that especially with young people we are in this stage whereby, I am also young, and I know that okay when I get a girl I will be counting times that after such time I am supposed to sleep with her.

Interviewee: Yes.

Interviewer: You see, so, I need to know again since it would be a good thing for me like I know it’s a need I want to have sex, but then when I do it, I want to do it in the right way, so that I protect the person that I am having sex with. So, if then I can get to understand how do you then avoid or how do you do it in such scenario, because some people I have come across say that there is nothing I can do because the moment that I tell the girl that I am living with HIV then she will leave me, so I will just have sex without telling her.

Interviewee: I think the funniest thing (mm) at some point, and I think the funniest thing is that, you know when you are dating (mm) you do not know each other’s status right?

Interviewer: Yes.

Interviewee: So, you might feel depressed and stressed too much (mm), but only to find that she also have one too, but you have been overthinking about it and she is relaxed (relaxed).

Interviewer: But don’t you think that it would be a good thing, like if you get to know that okay maybe she is also and then you can see like how can we.

Interviewee: It can cause a lot of conflicts.

Interviewer: Why?

Interviewee: You know like because having her to know that I am HIV positive, or for me to know that, she is HIV positive, it would be an argument that will happen at the end.

Interviewer: (Laughter) why you do it, okay like what kind of argument do you think it will come from it?

Interviewee: Why didn’t you tell me (mm), you know, she would have supported (mm) and some point they lie though (okay), and you know like let us go for a test (mm) you know and maybe I have it now, but you really know (okay) and maybe accuse you of infecting them.

Interviewer: So, let us say in your scenario whereby you have not had sex with each other, and she might ask you like okay why don’t we, or maybe those people might come to school, and she can ask you lets go and get tested.

Interviewee: In school?

Interviewer: Yes

Interviewee: Ey, no! (Laughter) you see that is the thing I am trying to say that it is support (mm) but they are trying to do good things (mm, mm), but at some point, but people doing tests in public (mm), they wouldn’t come

Interviewer: So, if it’s done

Interviewee: In private

Interviewer: In private? (Okay)

Interviewee: Yes, because when we come in public (mm), there is always people who are waiting to see

Interviewer: To see what’s happened, so in that case then, again let us say maybe then there is because sometimes they do those outreach that happens that sometimes they just put a tent, so if that happens that maybe she sees that when you are just chilling and passing by and say, let us just go and see.

Interviewee: No, I would not

Interviewer: Why would be your reason because it is not like you are in public now

Interviewee: I do not like it (laughter), or I would go but I would ask the nurse to tell me privately (mm) so yah (okay).

Interviewer: So, okay maybe there is something that you need to know, let us say that the tables are changed, and your girlfriend comes to you and then you are just chilling, and she says you know I did, I get tested the other day and I have just found out that I am positive

Interviewee: (Background noise) I think to me it would be fine, because I would have known that I have someone with the same situation (mm), but at some point, I will think about the future you know (mm), that maybe if we carry on doing this sexually activities thing (mm), they are chances of us staying much longer (mm), they are scarce.

Interviewer: They are scarce?

Interviewee: Yes

Interviewer: Okay, okay

Interviewee: So, it wouldn’t be a problem (okay).

Interviewer: So in other words you are assaying if it was up to you, you would want a partner who is HIV negative?

Interviewee: (Silence….) Yes

Interviewer: Okay, and…let’s say then eh…that is the case and it happens and you are not saying it now, but when you are quite mature and you haven’t really been having sex, you have been just dating, things are going well, you find uh..a job and things be like, you are getting to a point when things are like stable, would you then at some point get to tell her?

Interviewee: Yes, at some point I would tell her.

Interviewer: You would

Interviewee: Yes, because having a …… (Mmm) to me….it gives me the sense of closure that my kids you know (okay) can be bale to live and have someone to look after them

Interviewer: After them

Interviewee: Yes (okay) but if all of us are positive, we can die anytime and leave our kids and I don’t want them to have the same life that I did

Interviewer: Grow up maybe as orphans and be…

Interviewee: Yes, yes yes yes yes.

Interviewer: Okay, no I understand what you are saying, I understand what you saying. Okay but coming back then so do you have an idea of why I’m doing this?

Interviewee: Pardon?

Interviewer: I am saying do you kind of understand why I am doing this research?

Interviewee: Yes, I do (laughter)

Interviewer: (Laughter), because that was the question that is asked (yah, yah), like people are always talking about this so why then are we doing it

Interviewee: Yes, I do (mmm) I understand

Interviewer: Because like those are two important things that I would like to address, the first one being that okay social support, like are there people that you can talk to, are there people who are giving you positive support (yah) not that negative support when they say negative things and bad things, no but something that is going to make you feel like okay, there is hope, I can do this thing (yah) and there are no people judging me. That’s one thing, and then again, the second thing is like we want to see like how can we have, because like we all have people and our dream is to have that life partner who is ah this is going to be my wife and we are going to start our family (yah), so how do we go about doing it, you see that is what I want to see

Interviewee: Yes, I understand

Interviewer: And from what you have said like I’ve-I-I have seen, I have already got like a lot of information that I can just listen to it again and say okay this is what you were saying and it really makes sense (yah) it really makes sense

Interviewee: Okay

Interviewer: So do you feel like you have something to ask

Interviewee: No, I am fine

Interviewer: You are fine?

Interviewee: Okay

Interviewer: Thank you so much for that.

Interviewee: Yes, thank you

**Interview 9**

Interviewer: ….We will continue then from there

Interviewee: See like, it’s over the years (mm) I’m 23 now, like since I’ve been through ups and down, working here working there, but I’ve realized you know, sometimes what you want in life, its-it doesn’t really come as you want it, you know (yes) it doesn’t really come, or maybe it doesn’t even really come, buy you’ll find something else (mm) that matters to you, you know (mm) like maybe, its maybe not a career, maybe it’s not a job (a job). Maybe it’s even your family at home (mm) that keeps you in a drive you know (yah) carrying on, that’s my-my strength (strength)

Interviewer: Because umm..for some know some people, especially um..those people because I have come across people who have really not disclosed their status, especially to family and it becomes uh difficult for those people for their family to give them that support and drive, but I guess uh, in your situation as you are saying, you have your family who gives you that support, that drive..

Interviewee: I have told completely all my relatives about my status (mmm), I’m not afraid of them I’m not afraid of even who comes in at home, even if they ask me questions you know (mm) maybe I’m talking about my status if they walk in, I will carry on talking (talking) about what I’m talking about (mm) because it is my-if you want to go out and go and talk to your friends and neighbors, it’s your own story (it’s your own story) because you’re suffering from your own situation (situation, okay I understand)

Interviewer: (Coughing) (excuse me) I will go back as I have said that mostly what I’m interested in gatherings is like okay, how as a young person do you feel about your body, how does that maybe affect uh relationships. So, firstly maybe the question that I need to ask like as a young person who is probably uh living with a positive status, like does that affect you look at your body, how you feel about yourself?

Interviewee: Like, maybe with the opposite sex like?

Interviewer: No, before you even go to the opposite, like for yourself as an individual, maybe do you feel like the fact that maybe you are living uh with the positive status, does it change the way you look at yourself or maybe you feel like with your body maybe you-you are losing weight or you are not as fit as you want to be.

Interviewee: Yes, I-I do feel like that sometimes, but I’ve actually made a friend in my head, maybe you are going to say I’m off my head (laughter), but I’ve made a friend in my head (mm) who I talk to, I talk that to (to you, mm) like if you are talking to me and looking at me in a funny way (mm), I will talk to this person

Interviewer: In your head?

Interviewee: It’s my friend, my personal friend (mm) but maybe you will … say I’m off

Interviewer: But what do you-what do you talk to this person in your head?

Interviewee: About how I feel, I talk about my situation (mm) and how- and what do-you know like I know that I can do, like I tell myself positive things (okay) with this person (yah) it’s like me talking to me.

Interviewer: Yourself

Interviewee: Myself

Interviewer: Okay

Interviewee: And encouraging myself.

Interviewer: Oh, so basically things that you should be talking to your friend inside your head, mostly is like mostly positive things?

Interviewee: Positive things. And it encourages me even when I’m weak, maybe I’m down in one day (mm) like you see for …is a positive person (mm). like you see your emotions is for-its affect your health, so you see like maybe (mm) if you are sad and down and say yeah, you look sick, it drains you (it drains you) although you can eat healthy, you can but your emotional, even if a person maybe he upsets you (mm) to-to lowest you know it hurts your heart (mm) it’s like something inside you will just trigger the-the violence or maybe its within yourself and you feel sick

Interviewer: Ow, so it’s like anything that will happen like emotionally it gets to you physically?

Interviewee: But if you take it in, its-its, if you take it to your mind (mm) then it ends up hurting you, but if you find a way to separate yourself away from that atmosphere, I find my own atmosphere in my own mental state (state, mm) yes, I talk to myself

Interviewer: Okay, and you always try to give yourself like that positive feedback?

Interviewee: Yes.

Interviewer: Okay. So like, that’s about you and how you think about yourself. What about others like the way that others perceive you, do you think it actually affects you personally?

Interviewee: (Coughing) before it used to, like most of the friends like (mm) some-some small children would say they’ve know my status maybe their parents have been talking (mm), and come say you will die (mm) what, what, what, so you see, maybe I’m thin because you see symptoms of HIV positive will be looking thin (thin) and looking okay and they will be looking okay (okay), yah, they’ll come to you and say who you’ve got AIDS what what what! (mm) and you know what the child is saying it used to hurt me before, but I said yes I’m dying of it, I wanna die tomorrow too (you never know). Yah I told him-I told them straight you know (mm) because the-I’ll send that child a message it’s better to send them with a message that will also encourage them you know (mm) that you know what, I’m happy with myself, so why are you worried about me (about me, okay). That’s what I am, the type of person I have put myself into (okay).

Interviewer: So, in other words you are saying that previously of course it used to affect you, but you got to a point where by you had to accept your sta...

Interviewee: Yes, all of that

Interviewer: All, of that. You said like okay, this who I am, if you can’t tolerate me and if you can’t accept me for who I am then it’s your own problem

Interviewee: Yes.

Interviewer: Okay. I think its somethings that makes sense. So like on the overall, what would you say, would you say okay I am satisfied with the way that my body looks?

Interviewee: I won’t say that I am satisfied with the way, I wish that I could be fitter, I could you know (background noise), but I can’t, I can’t change somethings, sometimes I can eat as much as I want (want) but I can’t pick up weight (weight, okay) you see (mm)

Interviewer: Like does that bother you in a way?

Interviewee: Yah, it does bother me sometimes with my weight, you know (mm) I’m not picking up weight (weight), sometimes I look like-my age is 23 but I look smaller than my age (okay), you know and a person will tell me that I look smaller than my age (your age), sometimes a person will tell me you know “what’s wrong with you? (Mm) you look smaller than your age, you’re not right?” I will tell him yes I’m not right (mm). I just accept that right (right) instead of making it an argument, because if you make an argument (mm) it hurts you (yah, yah, I understand). If you try to object yourself (object yourself, yah) to something that is not (mm), that is true you know (true) they are telling you the truth (mm) so why would you object it (object to that).

Interviewer: Yah, I get that. And like uh as I was saying like previously as a young person, you are growing up, it doesn’t matter where you are, but at some point you start thinking of uh having a relationship, it can either be a boyfriend or a girlfriend, like have you thought about that?

Interviewee: Yes, I had had a girlfriend, actually girlfriends, but none of the relationships worked, because of you know, their parents you know, if their parents know something they will tell her to stay away from him, so I can see you know changes (changes) and peoples attitude change (mm) you know, so I just pull away from those people slowly I cut off I stop greeting you and I walk away (okay).

Interviewer: So, like uh with those uh?

Interviewee: What? Sexually I- I I’ve never slept with a girl (mm) that’s the truth, because I believe you know I never want to infect a person you know (mm) I never want anybody to go through anything that I’ve been through (through) in my life, I know what it means to go through being sick in and out of hospital (mm) you know.

Interviewer: But, like uh if because you said like uh the past relationship that you had like mostly the girls would try to pull away or they will show you uh a different attitude probably after the parents have told them, but uh do you feel like if you were to find someone and they don’t pull out and do all those funny things, at some point would you be comfortable to disclose your status with them?

Interviewee: Yes, I would, that’s-that’s maybe of the knowing the person first, you see because with an HIV person you can’t just disclose for anybody (which is true). I wouldn’t mind disclosing same time (mm), but you know it’s like you would a way you uh maybe you would tell people that I don’t interact with that … (mm) be like people that will strong write in my face you know (yes) and hug me (will not sure), although he will like me but he will be talking to people, maybe you are a person that is talkative blablabla blablabla (mm) you know, so if I maybe get to know you maybe I get to know your personality, so I even-if we got like we start dating, I first tell her lets be friends (friends), let me get to know you (mm), because I rather be your friend first, know you better (mm), understand you and understand your state of mind (yes) and who you talk to, who you interact with (mm) and your type of friends and how you are around those friends (around those people, yah)

Interviewer: Yah, I understand. It’s like uh you’ve said of course with time in the long run you will disclose to someone who you would like to be, but then uh, maybe if maybe you were give a preference like what would you think it would be easier maybe to date someone who also has a positive status or you would be okay with dating someone also with a negative status?

Interviewee: Eh, you see like people don’t really tell you their status (mm) so you will never know whether the girl is negative or positive unless maybe you go to the same clinic you meet (mm) you meet the same clinic (clinic) you know (mm) you know this person maybe you know from the time she was small that this person is positive too (mm) and you start to like them (them) you know (mm), but if a person is negative now, uh, for me I would get to know them like how I said (okay) before I tell her, but if the person is positive (mm) I would immediately tell her (tell her) you know I’m positive like you (mm) and I would like to get to know you (know you, okay)

Interviewer: And like uh I know why I asked this question because someone so was telling me that okay, I might be worried that if I find a partner who is also positive and uh we want to have kids and what are the chances that we will be able to see uh our kids grow if were both uh posi-positive, what do you think about it?

Interviewee: The chances…I-I won’t say the-the HIV medication is 100% because you see when a mother is giving birth to a child (mm) a child also can cut in when coming (process) out of the womb (mm) it depends whether they have a surgery (mm) that’s 100% negative (mm) they just..(it’s safe) cut its 100% safe (mm) they off the belly and take out the baby (the baby, mm), but if it’s coming naturally (mm) eh there is like small chance like maybe you can say 1% or 2% chance (mm) the child-that child would have a small infection (mm) you see of that virus (virus) in them (mm). Maybe you think they’re taking a medication and will be doing as a mother, but yah you can have children that are negative (mm, okay). So, I’ve- I’ve done my research.

Interviewer: On that? Okay, but for you-you-would you really, as you have said you wouldn’t really mind if the person as also have the positive status as long as you like the person?

Interviewee: Yes, and she likes me

Interviewer: If she likes you back, which is quiet important. Okay, so yah I think, I’m sure you have said something this before, but I just need to clarify like so in terms of maybe it’s you, you have uh there is a girl that you have seen and you kind of like her, like do you think, before like you approach her do you think about okay, maybe do you look at yourself and be like will she like me with the way that I look, like does that come to mind about how you look before you approach a girl?

Interviewee: It does, it does come to mind you know, and you know (mm) sometimes you get the girl she’s sitting right next to you you know (mm) and you can’t say nothing she will be staring at you, you know (mm) and like for me another- another guy you know it’s easier for them you know, but for me now I’m thinking in my head, if I start this relationship, now what if her parents know my status (mm) you know I’m thinking all of that (all of that things) it’s just in my head and if this girl is negative you know (mm) what if she embarrasses me you know in front of other-other people (other people) that know me (mm) other friends that know me you know (mm) the crowd that I hang around (with) and let me feel down, bring down my personality (mm) in front of them (in front of them). So, I-I for relationship wise it’s very hard for me (mm) I won’t lie (it is difficult) it’s very hard for me to just approach a girl, even if I liked them (mm), they are staring at me, even if a girl approaches me first (mm) like I won’t-a girl just approached me three months ago in the library, she said that she likes me (mm), but I asked her how old are you, she’s a bit younger than me, so I told her you know I like you but (mm) lets be friends, enjoy your life, find another guy because you know I’m not the type of guy for you (for you) that’s what I told her, I just told her that I’m not the type of guy for you (for you, okay).

Interviewer: I understand that. So like uh in terms of maybe with your family or friends, like uh uh the way that they look at you and your body like uh does it affect you maybe emotionally like the way that they look at you

Interviewee: Not my close family, they are okay with me (okay), but I got other relatives that when they look at me you know like (mm) they try you know some people they can be your relatives (mm) they can try their best to smile, to be happy with you (mm) but certain things you can see off their face (mm) you know they said that your eyes and your face does not lie about anything about you (mm). Like me, if you are looking at me you can be smiling and be joking at me (mm) but I can see whether you like me or not (or not, you can tell) immediately (mm) you know (mm), whether you are disturbed about me or not (or not) and I can excuse myself (yourself, mm)

Interviewer: In like yeah like that way that they look at you even if they are pretending to be smiling whereas you see that they are not how does that makes you feel?

Interviewee: It hurts, I feel it and I just keep it here (yah) but I know you know what, they are trying (yah) they are trying, they are my family they are trying, I understand (mm). They haven’t dealt with this before, but they are showing me better of what they can (what they can, okay). I have to just accept what my family can give me (can give you).

Interviewer: So, like uh, in terms of uh of course there are some people especially as you have said, you family gives you kind of positive they are supportive, while you might find that those other people they are family, they are not really close and they are not really supportive in a way.

Interviewee: Yah, I do..yah I do have both of that, so (mm) I’ve got an elder sister you can say, I don’t know, she just doesn’t like me (mm) I don’t know whether she likes me or not because, she’s I don’t whether it’s her own emotional feelings that she’s pushing on to me or what but she call me names that you know hurts, but (mm) it doesn’t hurt me because she is my sister you know

Interviewer: She’s your like uh…

Interviewee: Close relative

Interviewer: Close relative? Oh okay

Interviewee: It doesn’t hurt (mm) but it’s like…

Interviewer: But like when she calls…

Interviewee: But it’s like for a while

Interviewer: When she call the names like, what’s her intention?

Interviewee: Mmm, she gets angry for no reason, like maybe her intention would be like small stupid things, like maybe see like, I don’t like explaining about her maybe I will feel bad as I explain it as it is

Interviewer: Okay, no I understand, I understand

Interviewee: So, I don’t like to talk about her

Interviewer: No, it’s okay, it’s okay, mm

Interviewee: Yah, it hurts, but argues about small sugar things in the house like you know (okay)

Interviewer: But…

Interviewee: about other things I never done she will accuse me of it you know (yah, yah I understand), because I’m the only boy in my house, I’m hardly there in the house (in the house, mm) and see me there but (mm)

Interviewer: You hardly there

Interviewee: Yah.

Interviewer: I understand. Of course you don’t have to go into that (laughter) detail. But of course I remember like initially you were saying uh you have uh that kind of friend of voice that you have in your mind that gives you kind of positive feedback that gives you hope, but again I just wanna to know like okay, besides uh that voice that you have like, what other things or what kind of other mechanisms do you, what other things do you do, that keep you going, like that make you resilient to everything that will be going on around you?

Interviewee: I shut myself off, it’s like I’m standing here, I will look for an object (mm) like I’ll close it’s like my ears and everything is shut down (shut down) (laughter) I don’t know even if you know how to do that.

Interviewer: No, I’m trying to, because like in a way you are saying like you are blocking everything

Interviewee: Everything

Interviewer: Everything out, mm

Interviewee: See, I will be looking at you (mm), you will be talking now (mm), but I’ve learnt (mm) this type of attitude, I will be just be saying yes, yes, yes and I agree to whatever you say (laughter), but…

Interviewer: But you are no actually listening

Interviewee: I’m not there (mm) it’s like my mind is somewhere else, I’ll be still here but I will be in another place (ow) thinking of something else

Interviewer: So the person will be seeing you are physically there, but your mind is somewhere else?

Interviewee: Yes, and you would think that I’m listening to what you are saying (mm) even if you are accusing me or you are shouting at me for no reason (no reason), I will just be agreeing, and agreeing or maybe I’m there, but…and then you will get irritated by that (laughter).

Interviewer: Because you can see that this person is not responding, he’s not getting angry as well to what I’m saying.

Interviewee: Yah, you know people get irritated when you don’t get angry (angry, yah yah, mm). I don’t know, it’s just-it’s just a thing with people (mm) they know they are wrong, but they are shouting you for nothing, but they carry on and carry on, but me, I don’t know, maybe it makes a person more angry or what (more angry) but they end up having bullying up (mm) and they end up walking away (yah)

Interviewer: Yah, it makes them more angry because they are trying to make you angry as well but you don’t respond

Interviewee: To retaliate

Interviewer: Yes, it’s like what’s wrong with this person. Oh yah, I get it, I think I will be pissed off as well if it was me because I would be ah, but this person I’m trying to make him angry but is not like he is responding like what’s wrong with him and it even makes me more angry as well (laughter).

Interviewee: So, me I-I I have learnt that little lesson and you know a person can be doing that to me like even in a fight or something with somebody (mm) I have learnt to do that with people, where you can say anything to me (mm) it’s like I never hear you (mm) I block you out (okay)

Interviewer: So like uh even when people are saying hurtful remarks about your body or something about you, that’s-you have learned just to kind of ignore and block it out?

Interviewee: Block it out (mm) and it actually come up to you if they are maybe insulting you (mm) a person can come after you (mm) face to face (mm) and me I will carry on like I’m in a rush somewhere (somewhere else) I will run pass you (mm) and I will stop you and that there is …. In my life (laughter).

Interviewer: You will just try to just walk pass them

Interviewee: I will walk past you and this person will look like they are confused, and they are not going to say what they want to say (what they want to say).

Interviewer: And I think it’s a good thing because for you you will be just saving yourself the trouble of getting to hear whatever issues, because you-you would already know that whatever they want to say is not good for you, so by all means try to avoid it is the best way

Interviewee: ….. Because I got, I will tell you (mm) I do not have no friends now at the moment (coughing)

Interviewer: No friends of your age?

Interviewee: No friends of my age. I got a few people that I talk to, but I can’t really say they are friends (really friends) I can talk to you now, but you will run away (mm) you know I’ve notice you see when it hurts things or maybe you have something that will hurt, there is one person who is always there but he got his own he smokes weed, so I can’t ang around with people who smokes weed (yah, you can’t be…) he always, he’s already moving somewhere else where he want to get his (weed) his weed (mm) you see. So maybe you talk to him a little bit and (he goes) he goes and find his weed, so I talk with them, I’m friends with them but I’m not in the same (circle) circle you see (mm)

Interviewer: So but are you saying maybe previously or when you look maybe five years back, you used to have a lot of friends, but this has changed

Interviewee: Yah, I start cutting you off slowly, I watch how to react towards me (mm) how you … are but if you are using me, its fine but I’ll just leave , I will not even tell you anything (mm) but the I just stop I-I stay in my house, you call me I greet you (mm) and I ask what you want (mm), but my personal staff will stay with me (but..) I don’t give you what-what you want from me.

Interviewer: Like mostly what is it that they will want from you?

Interviewee: Like you see, I had this other friend, he used to come to me…just when I was in school (mm)… he wanted me to borrow him my games he will come home to play my play station and with his own, maybe he had the staff and this person with his friends is enjoying himself, you know they used to call me (call me, oh yah) so, they used to call me (eh) so I used to be like, oh okay that’s our life is

Interviewer: You only want to come when they need something from me

Interviewee: They need something from me, but now what I used to do now when you need me, even now with people when they come to me, there is other young-young by who is at my age (mm), when he-he’s-when I go to him and I go to his-his brother or what, also you know when we are going for a job you know they are working (mm) you know, there is work there, I used to call him, but they used to never tell me nothing (mm) they will…… (mm), so I said alright, let me cut away from them because that day you are partying and you are enjoying your life … (disturbances). (Coughing) He came to me uh…I think few months back (mm) he wanted a job, I was I was out of the job and he’s telling me lets go there let’s go there let’s go there. I’m wondering, I think I saw a long time you are coming in my house (mm) you worry-you are worrying me, your brother is working in a police force, you are worrying me now (mm, mm) I’m not working (laughter) but you worrying me. I said ey you know what, let me just have my heart, but this boy wanted to play around (mm) I can’t be working anywhere without my CV (mm) and you telling me that you are tired.

Interviewer: He wanted you to do for him?

Interviewee: Yah, you tired (mm) if you want something, you make sure that they want (mm, exactly) you can’t be sitting in the corner and smoking ….with your friends (mm)

Interviewer: (Laughter) and you are saying you want a job?

Interviewee: Yah

Interviewer: Ah, it doesn’t work that way

Interviewee: And I’ve got no job you asking me for a job (a job) (laughter)

Interviewer: Its funny (laughter). Yah yah its funny.

Interviewee: I even get a number, you see I have a contact (mm) of this other…he told me recently about some grass cutting and…but I don’t wanna go back because I had a quarrel with this man (mm) he’s got taxis and he’s got small business, but I don’t wanna go back there because we had a quarrel with them (mm). he will call me over WhatsApp, knowing that our professional way to do this is through a message (mm), but he will call a person through WhatsApp. Three days ago and I go, when I see the message, it came through my phone when I was at library (mmm) I was out of the job, after the contract ended (okay) I don’t have money now, he sent me a message through WhatsApp, I went to the library it came through (mm)…and the message came through now (oh, laughter)

Interviewer: But it was from way back?

Interviewee: he just told me you see your ….. but you can’t blame me (mm, mm) I know you the-you the boss here (here) but you can’t be making me like a fool and show me the message (mm) this is unprofessional (mm) you can’t send a message through WhatsApp (WhatsApp) and say that thing you have sent me a message, you called me (mm) through WhatsApp (WhatsApp)

Interviewer: Ya, yah of course it doesn’t make sense

Interviewee: You’ve got money to-to-to go into WhatsApp (mm) you can’t phone me (mm) and tell me to come down (to come through), you telling me-you phone me first, you sending the message first (yah, no) before anybody else, but you never come (come), you lost out, who lost (ay yah) and I said you know what, keep your job (mm, he was a bit rude). I met him one day, ey they kicked him out that contract (mm) he left it, the same man that I was supposed to go to (to go to, laughter), people were fighting with them (mm), he met me once and he got another tender for something else (mm) he said, “…grass cutting and what and what” (laughter) eh you must come there and you must phone me when you want eh if you need a job if you need anything, because he knows me I find him people to work for him (for him) for you to win a tender you need people (people exactly) or else they won’t give you the tender. So, I knew this man has still getting the tender (mm! mm) and he needs a number of people (people) so he has to show them that they got the people (people) already

Interviewer: There….once they give you the tender, you can start working

Interviewee: You’ve got the equipment, but if you don’t have numbers here of people and ID numbers (mm)

Interviewer: To do the work

Interviewee: And the phone numbers, then they won’t give you (mm, the tender). So he thinks that I’m stupid, I don’t know how tenders are, but he knows that I have got contacts that I can get, so everybody can get.

Interviewer: Yah, that’s the thing, normally you will find that umm people they wanna come to you, when they need something.

Interviewee: So, he wanted to use me (mm) let me work and use me too (laughter)

Interviewer: Yah shame, but you are still be looking for a job?

Interviewee: Yah I’m still looking (mm), but you know God is good, that’s my-that’s my grace (mm)

Interviewer: Even now I am saying God is good

Interviewee: But even with my-my….uh females that will…that mxm (mm) you know, because a lot of girls that are-that are do like…but it’s up for me to approach them (mm) that’s the only thing that…but..

Interviewer: Because you have said it though, but the difficulty is that you will be thinking of the future, you will be thinking mostly like okay, how do I talk to them, like how do I disclose uh my status. Isn’t it like the things that you think about like, what does the-okay maybe are some of the things that hold you back

Interviewee: Yah for me it does (mm) you know like as soon as like you see, as soon as a person hears you are HIV (mm) they say you’ve got AIDS (AIDS), they don’t say HIV

Interviewer: mmm they jump the gun

Interviewee: They don’t know the difference between HIV and AIDS (AIDS, mm they jump the gun), especially if you don’t have it (yeah) or you don’t know about it (mm), they say, like you see in those places, actually there is like 90% of South Africans they don’t know the difference between HIV and AIDS (AIDS).

Interviewer: Yah, but I don’t know why it’s like that because there is a lot of education that

Interviewee: AIDS is like your body is not ….to any disease

Interviewer: Disease and they are number of disease that are living

Interviewee: So they are pushing you like a contaminated area where you won’t get affected (mm), they separate you from civilization, they put you in a-in a hostile that’s where when the-when you have AIDS (AIDS, yes).

Interviewer: Which is different from HIV

Interviewee: HIV you can live like a normal person

Interviewer: A normal life, you can live as long as you take your medication on time every day, then you are fine

Interviewee: Like even to a working environment, you know if a person, if they find out in your (mm) working place, even a boss himself will treat you differently (differently), and you know it’s not nice for your boss to be treating you like you can’t do this (mm) you won’t do that (do that) or maybe emm put you in a different area compared to other people (other people) because of your status and maybe you got the education or not

Interviewer: You have the qualification, you can do the work

Interviewee: Like he will make you work cleaning there (mm) because he see you know what, this person here is HIV positive, he is already gonna die (mm) let him go…. You see? (Mm)

Interviewer: Yah, yah it’s not okay and like it is very unfortunate that it happens, but like it is also illegal for that to happen, it is illegal. There is no way it should be allowed to happen that way, but yah I just wanna say that I’ve learned a lot from you and I think it will be quite helpful for me when I’m doing this research. So eh-eh…

Interviewee: Is there anything else you wanna cover up on?

Interviewer: Ya, but I think you have already covered, but I just-I will just ask like uh in all in all like do you have uh maybe that one person that you say have this person, that I can talk to when I’m going through staff , I have this person who is always there to support me.

Interviewee: The only person in my life is my mother and if she has to go mxm, I’ll tell you the truth, my sisters will end up if-if I’m…if I don’t get a job and a stable career (mm) I would end up on the streets, that’s the truth, but like I won’t rely on my younger sisters (mm, okay). I’m-I’m-I’m-I’m, even though I am HIV positive and ill (mm) but I would end up on the streets (streets)

Interviewer: And like I am not sure of the relationship between your sisters, is that like your real sisters or

Interviewee: They are steps (steps), you see my-my one sister she’s-she’s actually staying in a boarding, or maybe if it’s not a boarding she’s in Pinetown with her husband, she is married, the younger-the elder sister (sister, mm), and the second one is staying with, with me in my room (mm) and my mother, she’s got a child (mm) and from her child’s father, she stays there but they are not together (mm). the father’s child is staying in the house at the moment, since they-my sister is not working, my mother is not working (working, mm) see now, but my sister-that same other sister (mm), the others, doesn’t like me (mm). so, you see what I’m saying, and I’ve got a small sister, she’s younger than me (mm) she’s still in school she’s in primary school (school, oh okay). So, now my situation is that I don’t have dependency (mm), I’ve got a step brother that he got the same situation, he got a child already (mm) he is bigger than me (than you). He’s got his own family (mm)

Interviewer: He stays in the place, is it a house or?

Interviewee: He stays in a house (ow), he’s actually he’s married, he has his own building (own building, okay okay).

Interviewer: Yah, yah I understand, I understand.

Interviewee: So, you see now, if go there by my step brother, how would it look (mm) you see

Interviewer: I understand.

Interviewee: So, I can’t go to my other family, my granny or my dad because it would be like I’m a burden to them (to them).

Interviewer: Yah, it would be a burden to them

Interviewee: And I have to get up myself now while my mother is alive (mm) that’s how my situation is now (is) and I’ve got dependency, no person I can rely on or talk to (background noise) I can talk to (your mother).

Interviewer: Yah, I understand, and I guess that’s the mis-uh at least as you are saying you have to get umm to pick yourself, so that you get umm something stable, so that you will be able to look after yourself and your mother as well.

Interviewee: (Background noise) That’s-that’s my overview of people that will like me (mm) as to get themselves financially and physically stable (mm) in life (yah), or else it’s hard to make it (yah, yah, it’s also difficult) it’s hard to make it in life, or else you will push from here to there (to there) and you end up got AIDS and then you end up that side (mm, dead) and you know people won’t treat you like, they don’t even those on medical organization (mm)…those organizations they don’t treat people right (people well, mm). They say they treat-they look like they treat you right (treat you right, but they don’t) they don’t at all (mm, mm). They treat you like you are dying or you are already dead (dead, mm).

Interviewer: And who would want to end up in that situation.

Interviewee: Because they know that you know what, your family doesn’t care about you.

Interviewer: So why should we.

Interviewee: They are getting paid (mm), they are happy its fine (okay).

Interviewer: Yah, but I just hope that uh in your case it won’t get to that point, at least I just hope it won’t get to that point.

Interviewee: Alright (mm) God bless you!

Interviewer: Thanks to you!

**Interview 10**

Interviewer: So, I said like the first question though we have to do again in your body, so if you can tell me that how would you describe how your body looks. When you look yourself like, would you describe yourself as like okay, my body is healthy, is fine, I like, how would you describe yourself, especially the physical self?

Interviewee: I would-I would describe it in…most cases (mm) in a good physical sense, yes sometimes there are times where you feel that your body has taken too much and has taken too much because you pushed too far in what you have been doing (okay), but in general physically it’s not like looking in a mirror and thinking eh…

Interviewer: It is not good.

Interviewee: It doesn’t look good, yah (laughter) sometimes you will think, you know like when you try and fit in something (mm) and it’s not fitting and you know it was fitting maybe two months or maybe a month (laughter) and you will feel like eish, no something is wrong (something is happening) yah I need to start you know (okay, okay) yah.

Interviewer: No, I understand. Okay that’s your own like thinking, okay when it comes maybe to how others might perceive you like, do you think like it affects how you feel about your body, like what others they think when they look at you?

Interviewee: But...the-the funny thing is like when you-when you ask somebody else , they always say you look the same, and then you yourself (laughter) you can feel obviously (mm) that you not the same (the same) but if you ask somebody they will say to me you are the same man (you are the same) unless maybe something is a drastic change (change) and then they will tell (they will tell) hey you know what, what’s going on (what’s eating you) to me (mm) you were right, something wrong (mm) yah.

Interviewer: So, in other words you are saying mostly is like something within your mind that maybe you feel like uh, I’ve a lost a bit of weight or maybe I’m changing, but the other person cannot actually see

Interviewee: It’s more like…over…the other person cannot (okay) the other person can-they can’t actually (notice it) they probably notice it once it’s very drastic (drastic).

Interviewer: Like okay, this like too much

Interviewee: Yah.

Interviewer: Mmm, but other than that…

Interviewee: Other than that they will just see you every day (the same person) or every week or every second month (mm) and today you are still the same. In some cases some people will be saying, you are actually looking fatter (fatter, laughter), but you think no it can never be (be, there is no way) Yah

Interviewer: Okay, I get it. So like in a general level, would you say though you are satisfied with your body, like how it is?

Interviewee: Yah, I’m satisfied.

Interviewer: You are satisfied.

Interviewee: There are certain areas that (mm) I would like to…maybe two sizes bigger or size bigger at least.

Interviewer: Which parts are those, I’m sure you are not talking about (laughter)

Interviewee: No, no no (laughter) no no no, it’s just like, maybe like waist (oh okay), because sometimes, like at the moment I’m maybe like size 28 (okay), so then sometimes when going out to buy clothes, it becomes difficult (difficult), because you can’t just get something, because most things that are…the style (mm) fashion has gone into whether you get your size that might not fit you at the bottom (the bottom, okay) and me being my height that I am (mm) and length wise it won’t be matching, it won’t be matching with the waist (oh, okay)

Interviewer: Probably it might be a size 30 waist

Interviewee: Yes, size 30, for the length is right, but for the weight is wrong, you understand (oh okay, I understand, I do understand, it makes sense)

Interviewer: Okay, so moving on like to something that I-I would thing that is more interesting like, especially for young people, we all know that at a certain point in time, you need to get on wake up and look for potential partners. Like before you actually maybe, get to approach her, do you maybe sometimes think about okay about yourself, about the way you do, the way your body looks, does it kind of affect to want to approach someone

Interviewee: No.

Interviewer: It doesn’t?

Interviewee: It doesn’t.

Interviewer: Okay, and again, it’s a good thing. So if you are about to eh...do you have a girlfriend or are you dating currently?

Interviewee: Yah, I have a girl.

Interviewer: Okay, so can you be maybe just tell me like how is it been like being involved in with someone?

Interviewee: It’s-It’s been normal, she’s been very supportive, and she encourages me, she can see you know you know (mm) I can see he’s-today he is not in normal self, because I a type of person that’s always joking and walking around and making jokes, so she can see you know things don’t look right (mm) then she will sit me down and see what’s wrong, what’s the matter (what’s the matter, mm)

Interviewer: Okay, like uh how is it like maybe disclosing to her?

Interviewee: It was, it was, it was- well the day when I told her (mm) the-the action was normal, she said it doesn’t change the person that I am (mm) because as well my parents also have it (mm), they’ve had it so (mm). When I explained to her and give her all the details, she said it doesn’t make a difference as an individual, and she said yes if you tell somebody else you automatically become labelled (yes) as that time of period (yah, yah) that person get to used pulling to (mm) and then she will start to look at you differently (differently), they will try not to do things with you (mm), but she was, she said things-things doesn’t not change (does not change)

Interviewer: And like uh how long has it after you have been dating for you to be able to talk to her. Was it maybe after a month or two, like how was it in the relationship?

Interviewee: It just like-like a few months

Interviewer: A few months?

Interviewee: Yah

Interviewer: And then you told her?

Interviewee: And then I told her

Interviewer: Then did like for you, before you told her, what was like your feelings your thinking?

Interviewee: Obviously, I was anxious and worried, like if I tell her, the reaction (yah) because that’s the main thing (thing, yah, yah) yah I was...I can say, can say scary and concerned because you don’t know (mm) how the person will respond, because most-most people are uneducated as for what is about, and people can talk (mm) the minute you tell them, the people… (mm, yah, yah most people) so that’s the first thing that comes to somebody’s head, they used to think, hey this person is dead (this person can die, mm) yah, so

Interviewer: Okay, and I like I wanted to also know, still on that of course you said like.., and like I just want to know that was part of that fear had to do with what if she rejects me?

Interviewee: Yes, part of it was and I think any-anybody would think that (mm) because on another given chance with somebody else, somebody else could have went out (yah) and could have said you know what I could have maybe just silence or not say anything (anything) I could have just disappeared, disappearing act (laughter) yeah.

Interviewer: Yeah, it usually happens, like it starts on…

Interviewee: Like why this person shying away, (mm) yah.

Interviewer: Okay, but it is a good thing like you said like she has been supportive throughout, lie uh okay, moving on to something a bit more intimate, like have you guys maybe consider or being intimate as like sexually?

Interviewee: It has gone to that point (mm) and we’ve making sure that we are using protection and she also goes on regular checks at the clinic, just to make sure we know (mm). And we have spoken about like should maybe there is something goes wrong or should in future we need to tell her how we need to go about like we do try to get a lot of information (mm) staff like that.

Interviewer: Okay, on that like uh, have you maybe sat down, just the two of you and say okay, if you wanna have children or maybe you have just gone to someone to talk to so that they can give you more information, how have you-is it like only the two just discussing?

Interviewee: It’s just the two of us discussing, or maybe she will do some research, she will-recently, not recently, it’s like a few month (mm) she bought a book uh…I can’t remember the title of the book, but in the book like it explains like the window periods and staff like that (okay) and how the whole procedure work and then I think there is also like medication for females that can be taken before even contracting (yes) mm, mm.

Interviewer: Yes, uh PrEP.

Interviewee: Yah, so (mm), she’s, she’s trying to be supportive but also educate herself (as well) instead of coming into it…and, and just like make it look like a…at least tell her what she needs to do (mm). She’s showing and...that you know what, I’m here with you (mm) we’ve…we’ll get there (get there), and then when the time is (okay).

Interviewer: And like uh, of course you’ve said you’ve been talking about kids, and like uh besides her coming up with the information maybe doing research on the internet and staff, do you feel like maybe there is uh enough information or there is enough places where you can get that information that you need, like for you to plan how live uh a healthy life and style, whereby you and her can also have healthy kids. Like do you feel like there is enough information or resources out there on that?

Interviewee: There is enough information, nut obviously, we will, we will need to speak also to somebody else, because just the two of us (mm) not being the medical practitioners or anything (mm) so at least if we can speak with somebody within a medical, maybe a nurse (mm) or not somebody that deals with in depths (mm) and then obviously that one person will explain thoroughly and will put the information that we have gathered with the information we got from the person and can ask (and kind of compare) eh and compare and see (mm) the way forward the steps that has to be taken on how we need to be taken (okay) yah (okay).

Interviewer: I get it, it uh going back uh to the fact that you said like uh, uh you mentioned that sometimes she can tell if maybe you are not, you said you are someone who is usually joking and staff (yah, yah) and she can pick up uh when you are not in that usual sense. I would want to know does she also pick up maybe when maybe you’ve lost weight or something is not right like with you physically.

Interviewee: Physically (mm) yes she does.

Interviewer: And then she does point it out

Interviewee: She will point it out.

Interviewer: How does that makes you feel?

Interviewee: (Silence) before, before I used to pretend like uh it doesn’t make me feel… you know (mm) like it doesn’t eat me (yeah) I would go home and then think on myself that something is wrong (mm), but now I, I actually use it as, what can I say (mm) as like more of a pillar, because like if she says to me ay no I can see hey (mm) depends on…you really gaining weight, you know what, there is something that I am doing right (mm, exactly) and then like, in there is an option is she will say ey what happened, like something that I you can, you can…(mm) looking like you are losing weight (mm) then I will speak to her, I will tell her you know what maybe I can stress upon this (mm) this is the problem, I haven’t been eating well, because of a, b and c (mm) and like staff like that. Like in the beginning when I started taking…uh when the medication was switched, it became a little of a process that every time I took it, it wanted to come out same time (okay), so it was making it hard for me to eat (mm), so what I would do is just eat and sleep (and sleep), eat and sleep, eat and sleep (and sleep, mm) and then she started to notice like the patterns and see you know (this has changed) you not looking good (mm) you looking like you tired all the time like what is wrong (mm) and then I explain to her and then you know at times there is nothing to be ashamed of (mm) if, if you need help that’s the reason why I’m here (mm), otherwise I wouldn’t have been here (yeah). So, if you feel that you need help in certain areas (mm), come to me speak to me, if I can’t help you, we can find somebody to help you (that will help you)

Interviewer: Okay, that’s good. Like uh from the way that you are saying, it seems as if like she’s been one of those people who’s been very crucial in being like kind of support system (yah) to you.

Interviewee: She has (mm).

Interviewer: And like uh, besides her like who else like is there anyone else like you can say it’s like okay I think uh these people of course have been really supportive

Interviewee: I would like to say my mother (okay) because she’s, she’s also been a pillar to lean I can say (mm) she always ask me are you okay, how you feeling (feeling, mm), like are you eating right, staff like that (staff like that, okay).

Interviewer: Okay, uh besides that like I know sometimes you might feel like maybe things are stressing and in life in general and maybe things are difficult like when you are faced maybe with those moments when you feel like uh it is too much, how do you manage, like what are some of the things that you do maybe to make yourself cope with?

Interviewee: Sometimes uh, what I will just say like if I can feel that I’m, I’m starting to get strained into I’ll just talk to-I will just sit back and realize that where I’ve come that’s far (mm) and I’ll first realize that I am fit, like I was continuous headaches, continuous fainting compared to now, now I’m…to playing uh football and won competitive events (mm) there is improvements and I always think there is always somebody out there that’s…maybe… (Mm) so me now wanting to throw the towel thinking that it is the end because I have reached a certain area (mm) that is unfair (okay)

Interviewer: So, in other words you are saying maybe when you feel like things are tough you kind of sit back and reflect

Interviewee: Just…

Interviewer: When you’ve come from

Interviewee: Yah, just, just reflect on how, how good, just to notice all the positives, although maybe, financial strain or whatever, physically strain (mm), just sit and, and I think of like the support system the people that I have, the number that you have (the good thing) and all that you still have in your life, because especially if you still have the parents (mm) most people don’t have parents (exactly) so all those things, you need to sit and think of all the things that you do have in life, instead of (the bad things that are happening), instead of…yes maybe you don’t have a job (mm) or things are bad at home financially or there is nothing to put on the plate or food (mm), but in order for you to cross that you have to sit and realize that you know, me cant sitting and contemplating and thinking about all the negatives that could go wrong with that wrong that has happened, as in that’s not gonna help (mm), because when you think of it (mm) thinking of something all the time won’t change, it won’t change what has already happened

Interviewer: It can even make it worse because of you thinking etc. (mm), okay. So like uh you said like now of course you have gone back to play football as well (yah), like uh do you feel that u okay that also maybe to stay, both physically to stay in shape and also emotional whereby you feel like you are part of something?

Interviewee: Yah it does, because when growing up I’ve been in a lot of…youth structures, a lot of famous youth structures that I’ve been there (mm) so when I was growing up (mm) I have been playing football (okay) I’ve left Durban to go play for my…and I came back then I went back to play into Johannesburg and played (mm), played…from one to here (mm, mm) and then from there once I started getting headaches and then I just lost interest (interest) in everything (mm) and right now I started feeling this is not right (not right). Things are not right I’ve started to fit into clothes like 26 and 24 (mm, mm) so, I’m thinking now ey things are not right here (mm) and then I was, I was later advised I think it’s like few days before my birthday I was advised you know to take test whatever (mm) and then from there, I actually…my mother that day (mm) and then from there like, basically it was only two options from this, it’s either you gonna give up (yah) that very moment, give up and perish and accept it because well how other people have accepted and say you know what this is faith so or you need to climb that area and make sure you move forward (move forward).

Interviewer: Okay, I understand, and like uh in terms of maybe uh physical fitness of course you’ve said maybe the biggest challenge has been like weight loss sometimes (yah) and like how are you managing to kind of…that?

Interviewee: Well, what I would do like as is said to you (mm), before it used to be hard like the eating and staff like that (yah), but now I can say I’ve started to gain an appetite where I, I’m constantly eating (eating, mm) not eating on a regular things, thigs that are right, so physically I can see there is improvement as compared to where I was (as before, mm) maybe it’s, it’s not where I want to be, but there is improvement.

Interviewer: So, of course but you know that with everything that’s going on there is a better chance of you to be where you want to be.

Interviewee: Definitely

Interviewer: Okay

Interviewee: Definitely

Interviewer: Okay, okay, okay I mean I’ve said quiet a lot, I don’t know like do you have anything else you would want to say add on that or any questions for me?

Interviewee: No, uh I don’t think I have any questions (laughter)

Interviewer: You sure

Interviewee: Yes.

Interviewer: Okay

Interviewee: Do you have any questions for me perhaps?

Interviewer: Nah, for me I think I have asked like because for me like the most important thing that I wanted to know is like when it comes to you, like do you feel that your body, how your body actually looks (yah) does it limits you in doing certain things, but as you have said that no it doesn’t, it actually for most people that I was talking to I found that disclosing was quite a hassle because of that fear of rejection (yah), but at the same time they are involved uh with people (mm) and like once you get involved with people as times goes on there are chances that they are going to be intimate (yah) but then it would be a good thing or it would be fair for the person that you are being intimate with to know your status (yah), but it is up to them to make an informed decision if they want to continue or not (not juts, not juts). And like in-with most people that I’ve been talking to like they have –they will be having difficulty in disclosing because of that fear of uh what if, what if maybe the time is not right (yah)

Interviewee: But as-as I said, I think it’s because of the actual habit (mm), the stereotype (mm) there it’s, it’s been…the minute you mention it, because even for, for example if you-you have maybe like a crowd of friends and you mention something about it, something that you are HIV positive (mm) the comments that come out of there (mm) so if you not mentally good to tell yourself you know what, what this people are saying isn’t really true (true) because people only speak about what they hear they hear saying (mm, yah, yah). So, example if let’s say …says ey no one (mm) and then you get it in the next four years (mm) or the next two years, let’s say the next four months you probably gonna die (die), but if you’ve actually been like engaging with somebody that dies out of it you will know that the person will tell you, it’s not about dying (mm) if you looking after yourself (mm) well…

Interviewer: You can live as long as you want

Interviewee: You can still live, people out there are dying from other things (mm) apart from this (exactly, which is true). Right now people are dying from cancer, but people do not stereotype cancer like that (mm, mm) and somebody who has cancer they say ah shame, (shame) shame, shame, shame, shame (mm yah), but the minutes somebody says ey you know what, I’m HIV positive (HIV positive), he becomes a suspect, people are disgusted, so why aren’t we disgusted with cancer the way we are with HIV (HIV) where cancer is taking more people (than HIV).

Interviewer: I think of course uh it also have to do maybe with the stereotype like, where I say most people they get HIV born, maybe they were busy sleeping with too many people (yah) but it’s not always the case. People they get infected through different ways, some they are born with it, some just want different that they heard, especially that issue of disclosing, who do not disclose the status and but that, that’s the thing that people don’t understand like there are various ways that you can get infected (yah) and at the end of the day you might find that it’s not your problem, but someone that do not (yah) who’s so upfront with you.

Interviewee: But I think basically it just brought some stereotype on the actual, the whole situation (situation) that’s what it does (mm)

Interviewer: People do not have that understanding (yah) what actually goes on (mm) okay. And I yah it is surprising especially considering that the amount of education, information that has been put out there, but then people they are still uh ignorant, like they do not want to accept it for what it is (yah true) mm, mm, they are ignorant to accept for what it is. But yah, hopefully maybe with time when they re-engage with people it will come to a point whereby people will be open-minded (hopefully) hopefully (hopefully) mm, hopefully. Mm thank you for your time.

Interviewee: Thank you.

**Interview 11**

Interviewer: Uh…okay Sne, I am recording our interview, but we start I would like to-I would like to ask how old are you?

Interviewee: Um…I am 18 years old.

Interviewer: you are 18.

Interviewee: Yes.

Interviewer: Okay, please free to speak both in IsiZulu and in English

Interviewee: Yah.

Interviewer: So, my first question, my first questions that I am going to ask you will be about the appearance of your body on how your body looks, and please feel free to talk. So, it’s basically about the thoughts and feelings of adolescents and young people who are living with HIV, on their appearance of their body. So, my first question for you is to ask how can you-how can you describe the way your body looks?

Interviewee: Well, according to my own views or according to my own opinion (yes) I see my body as (even in your own views), I feel my body is okay, it is healthy and has strong immune system (yah) so you can’t tell by looking at my body that I have a problem (eh) or I am living with this virus, because I am following health guidelines and healthy eating as well (eh) for your body to be healthy and for your system to be strong too (oh, okay) yah.

Interviewer: So, maybe in terms of your physical structure, how can you describe your body?

Interviewee: (Laughter) Oh no! (Laughter) aw like, I can describe is as a rich body you see (yes) and sexy you see (laughter). It is okay you see (eh) yes it is okay! (Okay)

Interviewer: Yah, as for being sexy (laughter), so what are your thoughts on the way your body looks?

Interviewee: My thoughts as in how?

Interviewer: Your thoughts, what are your thoughts on how your body looks? Like what do you think the way your body looks?

Interviewee: It’s just that I am okay, I am stress free (yah) I am maintaining everything in my life (mmm) um…yes!

Interviewer: So you have positive thoughts?

Interviewee: Yes.

Interviewer: Do you think that the way people look at your body or think about your body affects you somehow? Does it affect you?

Interviewee: No!

Interviewer: Why? Can you please explain it to me?

Interviewee: Like, it doesn’t affect me how people look at me (yah) um...how am I going to explain this, they won’t see me…can you please repeat the question for me.

Interviewer: Okay what do you think-okay does the way people look at your body, or think about your body affects you, or does it have any impact on how you feel, like does it makes you feel in a certain way, does the way people look at your body affects how feel towards your body?

Interviewee: It doesn’t make me feel bad (yah).

Interviewer: Why?

Interviewee: Because my body is okay.

Interviewer: It is okay?

Interviewee: Yes (eh) maybe (laughter) if it have a weak immune systems (eh) maybe that would affect me because people would think something else about me (eh)

Interviewer: Pardon?

Interviewee: Maybe if my body didn’t have strong immune system and it was weak, maybe that would affect me when people are looking at me because I would assume that there is something they are thinking about my body since it was like that (ow, okay).

Interviewer: So, no matter what or how a person can say anything about your body, it doesn’t affect you?

Interviewee: No, it doesn’t affect me at all because they know nothing about me.

Interviewer: And you know yourself?

Interviewee: I know myself and they won’t realize that (okay)

Interviewer: Okay, let me talk about your physical structure, like when a person is looking at you, like while you are just walking in distant (mm) does it not affect you?

Interviewee: No, no it doesn’t affect me.

Interviewer: What makes you not affected?

Interviewee: Because I know that she is just staring.

Interviewer: She is just looking?

Interviewee: Yes, she is just staring, she knows nothing, so she will be just staring.

Interviewer: Okay like, I am referring to something that the person is seeing, for instance, she is looking at you

Interviewee: Something she is seeing in my body?

Interviewer: I mean your body in general, like if a person is seeing me, she is seeing a short person, so does the way people look at your body affects the way you feel about your body?

Interviewee: No, it doesn’t affect me.

Interviewer: It doesn’t affect you?

Interviewee: Yes.

Interviewer: Why?

Interviewee: I don’t know how to respond here, I don’t know

Interviewer: Or maybe it’s because you know yourself, that you are beautiful and okay.

Interviewee: I know myself

Interviewer: So it doesn’t happen, that if a person is looking at you, you will feel bad that (because I’m like this) she is looking at me because I am like this

Interviewee: No.

Interviewer: Okay, I like that. I like that (laughter). So, are you satisfied with the way your body looks?

Interviewee: Yes, I am satisfied.

Interviewer: Can you please explain why are you satisfied?

Interviewee: Um…I am satisfied because (mm) I can do things that I want to do (mm) and my body is flexible and allows me to do those things.

Interviewer: Things like what?

Interviewee: Things like what?

Interviewer: Yes.

Interviewee: Like…sports for example (yah), like I’m too good in netball, okay fine, netball, swimming

Interviewer: Really?

Interviewee: Yah, so yes, my body satisfies me (laughter), it is okay (yah) yes.

Interviewer: Yoo…so you are active?

Interviewee: (Laughter) I am active, you can see.

Interviewer: Wow! I like that (laughter). So, another part of the questions are about significant people in your life, like your relatives and people who are significant in your life. Significant people, like their perceptions on how they influence how your body looks and attachments with them. Do you think that the way your family, friends and your romantic partner think about your body affects you in anyway?

Interviewee: Their thoughts in my body? When they see me?

Interviewer: Yes, on how your friends, family and your partner think about your body affect you?

Interviewee: No.

Interviewer: It doesn’t affect you?

Interviewee: Yes

Interviewer: Why?

Interviewee: My family will not affect me, because I know that they know well about me, they support me, so they wouldn’t do something, or they wouldn’t think something bad about me (mm). For example, that was my family, even my friends, there are few friends of mines (yah) who knows and are supportive to me, so they are always complimenting my body (yah). Okay, I have a boyfriend and he also knows, so…he is also supporting me so, yah

Interviewer: Oh, you have a boyfriend?

Interviewee: Yes I do.

Interviewer: Does he know about your status?

Interviewee: He knows about it too.

Interviewer: Okay, so how did you disclosed to him?

Interviewee: How did I disclosed to him?

Interviewer: Yes.

Interviewee: Okay, I don’t know but he was realizing it, I don’t know how though (mm). yah, I used to be…what can I say, when I have flue, I used to have some little scratches (mm) as if something has scratched me (mm) so when I wear these tops that are fashionable these day (yah, I can see them, laughter) yah. So when I’m with him, he will just ask all of the sudden, why do I have scratch marks in my body (eh) and I would say, ah it’s just some scratches, and he will say really, just scratches, what’s wrong with you (mm), you see when a person will ask you just like that like that, like what’s wrong with you? (mm). like it seems like he was already suspecting, and he took my bag and saw the pills inside it (mm), and he asked what were the pills for because he had no idea (mm) and I said it’s for flue, so..

Interviewer: So, you used to bring the pills with you when you are going to see him?

Interviewee: No, they were in my bag, when I was visiting home in Pinetown (ow), I used to pass through him first, then he would open my bag (oaky). So um…then there was these scratch marks, so he would ask what’s really wrong with me, you see (mm). okay fine, but I always wanted to tell him but I was scared (mm), but I had no choice but to tell him, because he ended up shutting off from me, because you could see the way he was asking that he knew that there was something wrong (eh), just that…

Interviewer: But he was waiting for you to tell him

Interviewee: Yes! (Eh). So, I kept delaying from telling him and I realized that things were getting more tougher between us and he was threating to leave me, so...

Interviewer: You mean he wanted you guys to stop dating?

Interviewee: He was leaving me (okay). No, like he was shutting off from me

Interviewer: Okay

Interviewee: So, then I had to tell him (mm)

Interviewer: So, when did you find out, how long have you-since when have you been taking your medication?

Interviewee: When I was 2.

Interviewer: 2 years?

Interviewee: Yes.

Interviewer: Oh, okay. So, you grew up…

Interviewee: Having it.

Interviewer: Okay. So it was not easy to disclose that to him when you guys dated

Interviewee: Pardon?

Interviewer: I mean when you guys started dating.

Interviewee: No! I didn’t tell him when we just started dating, I wanted to know him better, and then I told him.

Interviewer: Okay. So were you guys engaging in sexual activities and things like that?

Interviewee: No, that was the reason that pushed me to tell him, because he was busy insisting that we do sex (you do sex) and then I kept on saying, no we must wait, and because we have been dating for years, he would ask what are really waiting for (yah) you see (mm) and then I kept on saying there is something I have to tell you, and he would get more irritated when I said that hence I would finally say nothing (mm). So he used to send me a text on WhatsApp and it’s not easy to respond on WhatsApp (yah) so he asked we talk on WhatsApp. So I said to him, no I will tell you, he asked to call me and I said no (mm), and the he asked what is it exactly, so it then got to an extend that I had to tell him (mm). and I wanted to tell him face to face because you wouldn’t know how a person is going to react when you are telling him over the phone (yah), like it is better when you see him (yah) so that you will know how is he going to react

Interviewer: How his actions will be, mm.

Interviewee: So it came a time and I had to be confident and told him, oh it was hard, I was brave and I told him.

Interviewer: Was it over the phone?

Interviewee: No, physically.

Interviewer: Okay.

Interviewee: So, I was expecting that he would want nothing to do with me (mm). Oh the time he asked me, he was holding my hand, and he begged me to talk (mm), so I said…I wanted to talk but I failed (mm), and finally, I told him. I just told him that I am sick, I didn’t mention what exactly the sickness is (mm), I just said I am sick and then I let his hand go off mine. I didn’t care whether he liked to continues holding my hand or not (yah). So, I let him go and he cried (mm). So, before that, he kept quiet for some time (mm), and I was like, eish I have done a terrible mistake or something (mm), also kept quiet for a very long time and face an opposite direction because I felling guilty, so I faced the other direction (mm). So, then he said to me…he started weeping and he said to me “why didn’t you tell me all the time?” and then I explained to him that it wasn’t easy for me to tell you (mm) when we were still dating and two, the way we were fighting, you were not going to say anything (mm) because I was scared that if told you we would fight and things get tough (mm). And then he replies and says all people in relationships they fight (yah) you had to understand that (true). So, he held me close to him, after I have told him everything, he held me close to him and then I asked if he still loves me, and then I let me off my body (mm) because you may never know what is he thinking.

Interviewer: What makes you…what is it that makes you doubt him, because you have told him and he held you after he heard everything, so why were you rejecting him by that time, you feel…

Interviewee: I felt like he hated me.

Interviewer: What makes you assume bad things about him?

Interviewee: I also don’t know (laughter). Well he hasn’t do anything bad (mm) but it was the easiest decision for someone who has been told such thing, you can’t assume a good thing about him (yah) so I was expecting anything from him (okay, yah). So that is why I let him go off my body, little did I know that I would see the other side of him (mm), so he accepted it.

Interviewer: So what was the side of him you saw?

Interviewer: Kindness (laughter) I didn’t know he would accept me.

Interviewee: Mmm okay, okay.

Interviewee: Ah, should I carry on?

Interviewer: Yes, yes its fine (laughter).

Interviewee: I don’t know if I you want to ask other questions (laughter).

Interviewee: So, um…I let his him go off my body and he assured me that he will always love me (mm) then I said okay (mm). oh then, he asked me what was my sickness, really now, but I am too young for him to think that I would have gotten this thing it somewhere (yah) then I told him that no, I was born with it (mm) it wasn’t easy for him to understand that (yah), but as time goes on, he understood it, so I told him. However, my question was…I even asked the nurses here, why he was so relaxed when I told him (yah). (Laughter), okay, okay fine he oved me, but after telling him this, he then loved me so much (mm). so, I am staying with my grandmother at home, (mm) we are so closed and we talk about everything (mm), so I asked her about this thing, but I framed the question to her as if I was asking for a friend (yah) I asked her like that (mm), she also said the same thing why the guy was so relaxed, maybe he also have it too (yah) and I said that is also confusing me, because no matter how you love a person, (yah) you can’t relax this much (yah). So, yes, he was relaxed, then we separated, and he said he would call me later in the evening, then we left each other. After that, he was ignoring me on WhatsApp in the evening (mm), okay he just kept quiet and kept uploading sad dps and kept quiet, and I didn’t know what was happening (mm) until he sent me text on WhatsApp telling me that he was shocked (mm) and then I said, oh okay. And then he said yah, and he asked me to give him time to maintain this thing that I have told him, and then I said okay. Then it came a time where we were okay and everything was fine between us, he then went back to the issue of sex (mm) like, what’s your name?

Interviewer: I am….

Interviewee: Nompulelo (laughter), like Nompulelo I was confused after that, because I have told him already, but still he was insisting on having sex (mm), like which guy would do that after you have told him everything about this. I was expecting him to run away because he knows about this thing (yah), instead of that he continued insisting on it (eh)

Interviewer: So, when you say he was insisting on having sex, how did he wanted you guys to do it? protection or without protection?

Interviewee: Okay, he asked me that if we do sex, do I want to do unprotected sex, and I was like me, he is asking me, what is that? (Yah). Do I want protection or not (oh)? Oh no he didn’t say it like that, he said, if we have sex, will I not feel comfortable if…bear in mind he is asking me (eh) if we use a condom.

Interviewer: But that wasn’t up for a discussion, like it’s something he wasn’t supposed to ask you because you also knew what should be done when having sex

Interviewee: I don’t know whether he wanted me to say something else, but my answer was “it’s your life”

Interviewer: Yes.

Interviewee: If you don’t use a condom what are you expecting to happen (mm) I asked him that and he said no, the reason I asked you this question is that I didn’t want to make you feel like I hate you when I’m using it (mm), so I want to hear it from you. There is no way a person can just accept so easily, I was suspecting him (mm) but why would he hide when I have told him already (yah) that was my other question because he wasn’t supposed to relax that much.

Interviewer: Tre, he was too relaxed.

Interviewee: But, yah we have never done sex until now, because If I do this thing I will have to think carefully (mm), because I will have to consider the consequences he may encounter (yes), yes and I also have to face the, okay fine I have started a big challenge by telling him (mm) because it is not easy to have sex with a guy if you haven’t told him about your status (yah). So, I am also giving him time to think about this thing carefully (yah), so till now we are okay, we don’t have any problems, but I am still confused, and I even asked my aunties why he is so relaxed (yah) after you have told him about this (yah)

Interviewer: How long have you told him?

Interviewee: It’s been 6 years now…no! its 6 months

Interviewer: 6 months?

Interviewee: Yes

Interviewer: Yo! And he is quiet like…

Interviewee: He is quiet, but my friends whom I have told are advising me to go with him in the clinic.

Interviewer: Yes, I was about to ask about it that…

Interviewee: Hell no! it’s going to be like I don’t trust him

Interviewee: No, it’s not like you don’t trust him, but it’s something all partners must do and it’s a good thing, even if you didn’t know about your status, it’s a good thing that you guys know each other’s statuses, because having protected sex is not for HIV prevention only, but if you know your partners status you can make an informed decision, because you might find that you can fail to do that, and if you accept that, then you can also support him and he can also support you too. So, the best thing is to encourage him to go for a testing, you must consider that so that you will also know where he stands because now you are confused…

Interviewee: But, he didn’t force me to tell him, I did that voluntarily

Interviewer: Yah…

Interviewee: So, it won’t be easier for me to say boom! You too must go for a testing.

Interviewer: No, he is your partner, do you wish to know?

Interviewee: Yes, I wish to know

Interviewer: Then…

Interviewee: But wouldn’t it be....like what I have been thinking about is that he told me his secret (mm) which is different from this one (mm) but it’s a real secrete, so what I’m thinking of is that if he is HIV positive, why is he not telling me because his secrete is almost the same with mine (mm).

Interviewer: Maybe he doesn’t trust you

Interviewee: But his secrete is the same wit mine (laughter) ey, but…boys will confuse you sometimes (laughter), but yah, after that then, we ignored each other (mm) we ignored each other, because we are staying far from each other, so we had no time to chill, but he sent me a message on WhatsApp (mm) and told me…at least he still cares (mm) and he told me on WhatsApp that he knows that we don’t see each other, but I will always love you. Obviously, those people will just focus on something else because of the distance (mm), so I was like okay, and I also have that thing that I have told him (mm), but he showed me his other side and he said “I want to ask you something, but I don’t know if you will think I am disrespectful” (mm), that was via WhatsApp (mm) and I was like, what is that? And then he asked if I won’t be mad at him (mm) and then I was like no, and then he said “are you still taking your medication” (mm), my question was why would a person still bother, I don’t know maybe it’s how I think (mm), but my friends said maybe it’s his own way of trying to get back to you (mm), but he can’t do that because we are distant from each other, there is nothing that can happen to us you see (mm), like we don’t care for each other but he is still asking that (mm). so I was like what does this has to do with you (mm), oh I responded to him and said yes and then I said, your actions are confusing me (mm) because there nothing going on between us (mm) we are far from each other, and then his answer was like “the fact that I am far doesn’t mean that I should care about you (mm) I still, you! I still care for you and I will always be there for you”. So, I am always think that this guy was the one (mm) because if it was someone else, he would have spread the news, tell his friends, since we are staying away from each other, he would be like ah, that girl…

Interviewer: Have you dated someone else before him?

Interviewee: Yes, someone from here at Blue Roof

Interviewer: Okay.

Interviewee: Yes, someone here at Blue Roof, so …

Interviewer: It wasn’t easy?

Interviewee: Yes, it was not easy.

Interviewer: Did he know about your status?

Interviewee: That I was in love with someone else before him?

Interviewer: I mean the person you were in love with knew that…

Interviewee: That I was in love with this one?

Interviewer: No, I mean about your health status?

Interviewee: Since I got him here at Blue Roof, we have the same problem.

Interviewer: Okay, so you knew each other?

Interviewee: Yes, we knew each other, and that was a perfect wit that one from Blue Roof

Interviewer: So, were you guys doing sex?

Interviewee: He is also staying far so…

Interviewer: Okay.

Interviewee: He is staying at Umlazi so…

Interviewer: You were not able to do it?

Interviewee: We were doing nothing.

Interviewer: Okay, so does a positive or negative feedback from significant people about your body or oh how your body looks makes you feel?

Interviewee: Please brief me on that question.

Interviewer: Like, the feedback that you get from significant people, like saying Sne is this and that, if it is negative…

Interviewee: As in does it affects me?

Interviewer: Yes.

Interviewee: No, it doesn’t affect me.

Interviewer: How?

Interviewee: Because they are telling me the truth (yah), it’s not like they are saying I am losing weight, what’s going on (mm) are you sick (mm), no. if I can go back to what I have said earlier, my body is okay, I don’t have any problem, I have a rich body and I am okay (mm) (laughter). So, they ask me what is that I am eating that makes me gain so much weight (mm) that is why I am saying even the pills do not affect me and I also think I am doing what is right and I must continue with that (mm), yah.

Interviewer: So, you were talking about the positive feedback?

Interviewee: Yes.

Interviewer: What about negative feedback I they are saying nasty things about your body?

Interviewee: Things like, is she pregnant? (Mm) why is she gaining so much weight (mm)

Interviewer: How does that makes you feel?

Interviewee: It makes me feel bad, because I know that it is not true (mm), yah.

Interviewer: Okay, so do you think the way-does the way your body looks limits you from initiating relationship with people?

Interviewee: No.

Interviewer: Why?

Interviewee: It doesn’t limit me.

Interviewer: Why, can you please explain to me.

Interviewee: Because I do not have like…how am I going to explain this, I don’t know (laughter) I don’t know how to explain this.

Interviewer: You can explain anyhow you want to explain it.

Interviewee: Please repeat the question for me.

Interviewer: Okay, dos the way your body looks limit you from initiating relationships with other people, and then maintain those friendships, for example, with friends or romantic partners.

Interviewee: It doesn’t affect me, because they would think that I am the right person (yah), and they will also think that I would do, I will help them with something else, perhaps that they cannot do by themselves, so maybe I could help them (mm), maybe because of my body as I am like this you see (mmm) (laughter) yah.

Interviewer: So, don’t you think that the way your body looks can limit you from making relationships with certain people or a person?

Interviewee: No.

Interviewer: And then after making those relationships, you will then feel like you don’t belong to those certain type of people?

Interviewee: No.

Interviewer: You have never had such feeling?

Interviewee: Not at all.

Interviewer: So, you are always okay

Interviewee: Top notch (laughter)

Interviewer: Do you think significant people in your life have an influence on how satisfied you are with your body.

Interviewee: What?

Interviewer: Significant people in your life?

Interviewee: Who are important?

Interviewer: Yes, who are important in your life.

Interviewee: Yes

Interviewer: Do they have influence on how satisfied you are with your body?

Interviewee: Yes

Interviewer: How?

Interviewee: Through their support, motivation and treating me well (yah) so yes.

Interviewer: So, does that makes you feel more satisfied?

Interviewee: More satisfied, yes

Interviewer: On how your body-your body looks?

Interviewee: Yes.

Interviewer: Okay, we are about to finish with our interview, and he last part of the questions will be about strategies that are being utilized by young and adolescent people living with HIV to negotiate a positive body image. Like there are many things people talk about your body, so the following questions will be asking how do you manage to deal with those things, especially those negative feedback, things you don’t like. So, how to manage to deal with it and carry on in life. So, how do you manage to accept your body as it is? How do you manage your body the way it is?

Interviewee: I tell myself that this is how it is, it won’t change (mm), um…and self-esteem

Interviewer: How does self-esteem helps?

Interviewee: Believing in yourself (yah) and knowing yourself (yah) um…yah!

Interviewer: Okay, so how do you manage to deal with hurtful remarks from people about your body?

Interviewee: Well…there is not much that you can do (mm), because you know that your body is not the way people think about it or see it (mm), so you just have to prove them wrong

Interviewer: Do not let them overpower you.

Interviewee: Yes, do not let them

Interviewer: Okay. so, do you have people in life who listens to you when you are talking and gives you support when you need it?

Interviewee: Yes.

Interviewer: Who are those people?

Interviewee: My family and my friends.

Interviewer: How, what kind of a support do you get from them?

Interviewee: About my body?

Interviewer: I mean yourself in general, your body and everything.

Interviewee: The support me, they tell me that I should always take the treatment (yah) they motivate me. They also show me other people’s experiences who have the same situation as mine, but they have made it in life (mm) which means that it is not the end of my life (mm), I can keep living this life (mm) it’s up to me how to live it (mm). So, there is that support that I get from my family and friends (mm) yah.

Interviewer: Okay, going back to your love life, since you have mentioned that there was a person you were in love with or people you have been dating, so I f I can ask for future, how are you willing to practice sexual intercourse? For example, if you fall in love with someone who is positive or negative, how do you want to do sex?

Interviewee: I don’t know.

Interviewer: Why?

Interviewee: Because I haven’t done it.

Interviewer: No, I mean will you prefer protected or unprotected sex? That was my question.

Interviewee: Unprotected.

Interviewer: Unprotected? Why unprotected sex?

Interviewee: (Laughter) Its obvious! And if you know each other (yah), and you have been dating for a long time, what are you hiding then? (yah). You must do it and enjoy it (laughter)

Interviewer: What if your partner is negative?

Interviewee: He will tell you, if he is negative, you are in love and you have got married (yah), then what is the problem with that, you too should be negative. Or you mean is he is sick too?

Interviewer: No, let me say he is not sick.

Interviewee: And you are sick?

Interviewer: Yes, you are sick.

Interviewee: Okay, and you are married?

Interviewer: Yes.

Interviewee: You must also get sick.

Interviewer: Wow! (Laughter)

Interviewee: We are fine after all.

Interviewer: You are okay, so there is no need for protection.

Interviewee: Not at all (laughter).

Interviewer: Don’t think that would be putting his life at risk?

Interviewee: No, it means my life would be at risk, where I am today? which means you are also going to be fine

Interviewer: So, you do not support using protection?

Interviewee: No, no, no! I don’t want that life! (laughter)

Interviewer: Okay, let us say you are both sick then?

Interviewee: Like we are all HIV positive?

Interviewer: Yes, both of you

Interviewee: How?

Interviewer: I am asking if you will use protection or not?

Interviewee: Both of us?

Interviewer: Yes.

Interviewee: If we are both sick?

Interviewer: Yes.

Interviewee: Hell no! we won’t use it.

Interviewer: You won’t use it, even if you are married or not married?

Interviewee: If we are not married we can use protection.

Interviewer: Okay, what about when you are married?

Interviewee: No!

Interviewer: Don’t you think that it can worsen the sickness?

Interviewee: (Disturbance)

Interviewer: Don’t you think that it can make things worse like becoming too sick

Interviewee: But how? Aren’t we the same?

Interviewer: Yes, but research says, even if you are the same, but your immune system is not the same, so you may find one of you has a weaker immune system so…

Interviewee: So, it won’t balance

Interviewer: Because our immune system is not the same, one of you might be affected...

Interviewee: No, no, no! So, what can stop us then if both of us have a strong immune system, as the nurse that I am coming from told me that my file says LTD and if both of our files says the same thing (eh), which means the virus is invisible as it has been explained to me (mm) so both of you have the same file, is there anything that can make you sick, I am just asking?

Interviewer: Ey, I-I don’t know on that…

Interviewee: You don’t know on that?

Interviewer: Yes.

Interviewee: Okay, I get you.

Interviewer: So, in other words, you mean you don’t tolerate using condom?

Interviewee: (Laughter) not to everyone though.

Interviewer: So, you mean once you get married?

Interviewee: Yes.

Interviewer: So, are you dating currently or?

Interviewee: I am dating.

Interviewer: You are dating?

Interviewee: Yes.

Interviewer: Are you dating with the one you got here at Blue Roof or?

Interviewee: Yes, here at Blue Roof.

Interviewer: Blue Roof. So, you guys are okay, and you understand each other.

Interviewee: Yes.

Interviewer: Uh…Sne I think we are done, I don’t know if there is something you may like to ask or add

Interviewee: (Laughter) Nop!

Interviewer: You don’t have any question?

Interviewee: No! I think it’s enough for today (laughter)

Interviewer: Okay.

Interviewee: (Laughter).

Interviewer: Thank you so much Sne, that was so helpful.

**Interview 12**

PN: How would you describe the way your body looks?

SM: Im okay, my body looks good. Im slim mostly and short.

PN: How do you feel with the way your body looks?

SM: To be honest I don’t feel comfortable, I just always feel like if I just gain much like just much more coz you know in society they think if you are slim you are sick even when I have to wear something I always have to see myself in the mirror and I’m like nooo, I just feel uncomfortable with my body because im slim. I feel like I just have to gain at least 10kgs and then I will be comfortable with my body

PN: Does the way other people perceive you affect how you feel about your body?

SM: Yes it does a lot. Because before I came here, obviously I was thin but not like this much. So I have lost more weight so every time they will be like you just small you so slim and that affects because I know that this is not my body and I know its because of stress and everything, the environment im in. every time they say comments on my body it affects me a lot.

PN: Are you satisfied with how your body looks?

SM: As I said before im not. For now im not.

PN: The way significant others perceive you

SM: My mom always makes sure that she cooks for me and make sure that I eat. Obviously she knows that it affects me but she will makes that I always eat and when I gain wait she will like woow that’s my child I know so she will be like I thought you were dying and she will be like I was so scared and it just becomes a joke. And my friend, the friend that I have she knows that like in terms of weight im so sensitive so supports me. She will be like you are fine don’t be like this just wear this, wear this like she always encourages me. My partner he also encourages me, he doesn’t give me that stress he gives me positive feedback.

PN: accept/reject you?

SM: Ive never felt or experienced rejection or discrimination but it is just in me who feel like I don’t fit in because I when I got out with my friends they are thick and all the stuff and im the only one who’s thin there. So its something that’s within me not that they say it.

PN: how does feedback make you feel?

SM: positive feedback makes me gain confidence like I feel its okay im fine theres nothing wrong with me im just okay. In terms of negative feedback it affects me it really affects me because if they comment I don’t respond at all but it affects and I keep it like that. But this year I have told them guys I don’t like what you are saying because it really affects me then after that they just stopped it. But when they used to say it, it hurt me because the body is very sensitive especially for women.

PN: Does yr body limit you in r/ships

SM: Ive never experienced such thing that my body is the one limiting in a way. It is just in me. That’s what I have noticed that the way I look at myself is not the same way others look at me (how do you see yourself?). I see myself like im slim and theres nothing interesting about me, im just boring like im that kind of a person and the other people don’t see me like that, they see like im sexy and all that stuff. They don’t just see me the way I look at myself. They see me as okay, sexy and all that stuff. So I always ask myself what do they see in me, what do I have. But my friend has told me that im intelligent, like I look like I can be wife material. So they see more in me besides the body.

PN: do significant others influence satisfaction

SM: Yeah sometimes, like it helps me before I had low self-esteem but now at least im starting to accept myself the way I am because they say positive things about my body and I starting to gain confidence because even when I take pictures I never used to take full body pictures I always take selfies but now I can take it and put it on my status. So im working towards building self-confidence.

PN: How do u accept your body as it

SM: I just accept myself as I am. Like it doesn’t make any sense to be stressed about my body. I also try to be positive and socialise with people who have positives thoughts.

PN: How do u deal with hurtful remarks

SM: I used to keep quite and cry a lot but now if u say something bad about my body I will just say so what. Theres nothing I can do because this is the way I am so I have accepted the way I am and im working towards fully accepting myself.

PN: Support

SM: Yes, they are my friend she does a lot and my family obviously. In terms of partner yeah but not always.

SM: I do value my body because it is a gift from God.

**Interview 13**

Interviewer: Okay Wendy, thank you for joining me, so before we start can you please tell me your name….ah, sorry, your age, how old are you

Interviewee: I am 21

Interviewer: 21?

Interviewee: Yes.

Interviewer: Oh, okay, so my question is about thoughts and feelings of young and adolescent people who are living with HIV on the appearance of their body, so my first question is to ask you to tell me how do you describe your body the way your body looks?

Interviewee: (Laughing) my body eh, to be honest I do not like it.

Interviewer: Eh, how?

Interviewee: It’s not because I do not like it, but since I have been like this...since I have been sick I do not feel comfortable

Interviewer: Please speak a little bit louder

Interviewee: I do not feel comfortable with my body. I see...eish, what I see in myself (please come a little bit closer) wat I see about myself is that when I have lost weight, I get stressed a lot

Interviewer: When you have lost how?

Interviewee: Wen I have lost weight (yes) I get stressed a lot and it doesn’t sit well with me, I feel like I could gain weight so that it won’t be obvious that I am sick, you see things like that (yes).

Interviewer: Okay, so like maybe if you can explain, how can you describe the way your body looks in general? Like its structure, what kind of a person you are or how do you see yourself?

Interviewee: Someone who is long (yes), sometimes who does not fit in because of her height (laughing) it’s the height, the height

Interviewer: That is a problem?

Interviewee: Yes, like when I look at my height and my age it doesn’t corresponds.

Interviewer: Oh, okay, I get you. So, what are your thoughts on how your body looks then, like what are your thoughts the way your body looks?

Interviewee: Ey, shame, I-I do not want to lie, I am not proud of it, I-I…you see I am not feeling myself and if I may be asked whether I am comfortable with my body, I am not comfortable

Interviewer: Why?

Interviewee: I do not know too.

Interviewer: Yah, but you just don’t feel yourself?

Interviewee: Yes, I do not feel myself, since-since I am sick eish. Ever since I got sick, I do not know whether it has an impact or what, because I had no problem with my height before (yes), but I just don’t like it.

Interviewer: Do you think it has an impact too?

Interviewee: Yes, it has an impact, because I am unable to-to accept it.

Interviewer: Okay, so you are not able to accept yourself well?

Interviewee: Yes.

Interviewer: Okay, I get you. So, does the way other people perceive your body affect how you feel?

Interviewee: Yes.

Interviewer: How?

Interviewee: (Laughing) Eish…if someone keeps telling you one and the same thing every time.

Interviewer: Same thing like what?

Interviewee: You are tall (yes) you are always tall, you see even if we are going out, because I have short friends, (laughing), so when we are going out, they are always saying, you though, don’t you get tall people like you, you see that thing (yes, yes). You may find that when we are going, you find yourself feeling uncomfortable on your dress code, because you are tall, and there are things that I do not wear because of my height (yah).

Interviewer: But you do like wearing them?

Interviewee: I do not wear short dresses (yah), I do not wear shorts (mmm) because of my height, and the fact that if you are wearing a high heel people will start commenting that you are too tall, but you are wearing a heel (ummm), you see those things. They will say you are too tall and whatever you are doing they will comment on your height, like you are dancing while so tall, you see things like that (mmm, mmm), so I end up not feeling myself.

Interviewer: You do not feel yourself?

Interviewee: Yes.

Interviewer: Oh, okay, so are you satisfied with how your body looks?

Interviewee: Not exactly.

Interviewer: Not exactly? Can you please explain (laughter)

Interviewee: I can say that the feedback that I get from people counts a lot (yes). As much as this must start from you or know yourself, but feedback and comments from people (yah), especially for me because I do not have confidence in myself (yah), so if…

Interviewer: You do not trust yourself?

Interviewee: So, if you are going to tell me that your body, you see, your height you see (mmm) that is going to have an impact in me because I do not have that thing of ignoring what others are saying.

Interviewer: You do not have confidence in yourself?

Interviewee: You see, I do not say I am okay with my body, no! I am not proud of it, so that too has a significant impact in failing to accept my height and to love my body the way it is, I can’t say I am impressed with it, no, I do not have that feeling.

Interviewer: You do not have that feeling?

Interviewee: Yes.

Interviewer: Okay, I get you Wendy, so okay the following questions are about people you are related to or significant people in your life, like how do they…perceptions of how body image influence attachments with significant others in your life, so my first question will ask does the way your family, friends or partner think about your body affect you in any way?

Interviewee: No.

Interviewer: Why?

Interviewee: Wat can I say though…my family, my family (ummm), because we are all tall at home (okay), so yes. However, my mother doesn’t like when I keep complaining about my height and she always tell me that I am okay, I must look at my brothers, since I am the only girl at home (yah) I have two brothers and I am the youngest (yah). So, it feels like if there was another girl, not boys only, because being tall is common in males and people can understand that.

Interviewer: It’s understandable. Are you the only girl at home?

Interviewee: Yes.

Interviewer: Okay

Interviewee: So, eish, they do not mind, they do not have a problem

Interviewer: Okay, so it does not affect you at home?

Interviewee: Yes, I do not have any issue.

Interviewer: Okay, you do not have any problem?

Interviewee: They always encourage me to love myself, and even if I do not feel myself, like I do not feel the outfit I have worn (mmm) my brother tells me that even if there is something wrong he sees in my outfit, he won’t tell me there is something wrong because I will lose my confidence, so he says I am perfect the way I am (yah), so he says I must just go, I am okay and even if I like a heel, he says I look good on it (yes).

Interviewer: So, your family is more supportive to you?

Interviewee: Yes.

Interviewer: So, what about friends?

Interviewee: When it comes to friends, eish they will always remind you that you are tall, why are you wearing such short thing (yah), wow, so you can also wear a heel (eish) and that irritates me the most because I wouldn’t have been wearing it if I had no size for it (yes, there would be no size for you) so, I just tell them that as long as I have a size I will not stop wearing a heel, so you will always receive such things from friends (yes).

Interviewer: Okay, I get you. So, what are about your romantic partner, do you have a boyfriend perhaps?

Interviewee: I used to have him.

Interviewer: You had him?

Interviewee: Yes.

Interviewer: And now you are?

Interviewee: (Laughter) He had no problem?

Interviewer: He had no problem?

Interviewee: Yes.

Interviewer: That your body was…did he liked the way your body was?

Interviewee: (Laughter) He was short, shorter than me, so I have never heard him complaining, even though he used to say that I was tall, but he was just kidding because we would laugh over it (yah) not in a serious manner, so he had not shown any problem with me because he is shorter than me, I was the one who had a problem (yah) because I asked myself what are people going to say when seeing me dating a guy who is shorter than me (yah) (laughing), so its awkward to date a short guy while you are tall, but he didn’t have any problem with that.

Interviewer: So, I would like to ask whether your family knows about your situation, like your health status?

Interviewee: No, it’s only my mother who knows.

Interviewer: It’s only your mom who knows?

Interviewee: Yes.

Interviewer: Why don’t you tell the others?

Interviewee: (Laughter) others…oh it’s my mom and my aunt

Interviewer: Who knows?

Interviewee: The rest do not know yet (mmm), and I do not stay with my dad (okay), but my mom knows.

Interviewer: Where does your dad stays?

Interviewee: He stays near home, but due to some circumstances they got separated with my mom (oh, okay), so my mom has her own house (okay). She stays with my three brothers, then there is…then it’s me and my mom (okay). My aunt also stays near home too, we are neighbors.

Interviewer: Oh, okay, so people who knows are your aunt and your mom?

Interviewee: Yes, my mom and my aunt, then my other aunt but I cannot count her because she lives far in in Eastern Cape.

Interviewer: Oh, but she knows too?

Interviewee: Yes.

Interviewer: Oh, okay, then what about friends?

Interviewee: No one

Interviewer: No one knows?

Interviewee: Yes.

Interviewer: Why don’t you tell them or talk with them?

Interviewee: I like to talk with them but the only person that I can share with lives far from me.

Interviewer: A friend?

Interviewee: Yes (yah), the rest are friends but I can’t.

Interviewer: Why?

Interviewee: They talk.

Interviewer: How?

Interviewee: They judge a lot

Interviewer: Oh they judge a lot?

Interviewee: Yes, they judge a lot and they can’t keep a secret

Interviewer: Oh okay.

Interviewee: When a person talks, when a person…you-you-it feels like once you have told a person there will that thing and they will be always talking about that topic so you will not feel comfortable, they will show you some pity for no reason and I do not like that thing

Interviewer: You do not like that self-pity

Interviewee: Yes, I hate self-pity especially about this sickness, I do not have a problem but when people are always talking about HIV positive people, HIV positive people, it makes me not want to disclose about it (yah). So, since they do not know, we are now able to talk with my friends and I do not mind when they are talking about it (yah), but I have that thing that if I may tell them one day, there would be a problem.

Interviewer: So, you can’t tell them?

Interviewee: Yes, I can’t tell them. The only person that I would like to tell lives far, but I am about to tell her (okay).

Interviewer: So, for how long have you known about this situation you are going through right now?

Interviewee: Ummm…20-2014.

Interviewer: 2014. For how long have you known your friends?

Interviewee: Ummm…ey

Interviewer: For a very long time?

Interviewee: Yes.

Interviewer: Okay, but still you do not want to tell them, and you are not even willing to tell them, except the one…why do you wish to tell the one who is far from you?

Interviewee: I am too close to her (yah), she listens to me when I am not talking, and she doesn’t judge, and I know her she doesn’t have many friend (yah) you see that thing? (Yes). She doesn’t have many friends and I know that me and such person are her friends (yah), even though I am not a friend to that girl she is a friend with (oh yah), but I know that you can talk to her about something (yah), you can talk with her (yes) and the rest ay no.

Interviewer: You cannot talk with them?

Interviewee: If I can talk with them, I should be ready that…

Interviewer: You are ready tell the whole world?

Interviewee: Yes.

Interviewer: Oh, okay. So, does your partner knew about this, have you told the one you were in love with that…

Interviewee: He knows.

Interviewer: Oh, he knew?

Interviewee: Yes, he knew.

Interviewer: Okay, so how was he, like was he negative or?

Interviewee: To be honest I do not…. I think he is positive.

Interviewer: How, why do you think he is positive?

Interviewee: I think he is positive because when I was in love with him, I know what kind of a person he is, I knew what kind of a person he was.

Interviewer: How?

Interviewee: He likes girls too much.

Interviewer: Oh, he likes girls?

Interviewee: Eh-eh…how can I explain this, he used to like girls (okay), but I was not sure because I was not living close to him (okay), but I knew because when I searched his phone I knew about it (yah), so this thing happened and I found out that I was sick…oh before that, we started by having sex (laughter).

Interviewer: You had sex?

Interviewee: Yes, we had sex, and after that I got sick (mmm), when I was sick I came here, my mother brought me into this clinic.

Interviewer: Here at Blue Roof?

Interviewee: Yes, at Blue Roof.

Interviewer: Okay.

Interviewee: And then after that, after that I then told him that I was in the clinic and one, two and three happened, I am this and that (yah). He didn’t have any problem, instead he comforted me and said sorry, sorry (mmm) and then he told me not to tell anyone about this (wow!), he told me not to tell my friends that I am sick and then I said okay I will not say anything to them (mmm), I will not say anything because I know what kind of people they are and he also knows them because I always tell him about them, then he said okay and I kept quiet. I kept quiet for a very long time and I finally asked him why he doesn’t want to go for HIV testing (mmm), you see we were just chilling together and he was telling me about his issues with girlfriends by that time.

Interviewer: His girlfriends?

Interviewee: Yes, and he was complaining about them, because we were too close to each other, so he was being open.

Interviewer: You were like friends?

Interviewee: Yes, and he was the first guy I met in my life (ow).

Interviewer: So, before you had sex with him, you were not sick or….

Interviewee: Yes, I was okay

Interviewer: Or you haven’t been tested before?

Interviewee: I was not sick, I tested before, my mother got me tested, and we tested with my mom.

Interviewer: Oh, and you tested negative?

Interviewee: Yes, I was negative.

Interviewer: Okay

Interviewee: I tested at Mshiyeni.

Interviewer: Alright.

Interviewee: So, as time went on I started paying attention to what he was talking, and he is always sick (yah), and then I asked him why he didn’t want to go for testing at the clinic?

Interviewer: So, in terms of him being sick, did that started before you knew about your HIV status or was it after you knew about your status?

Interviewee: No, he used to get sick and he was always complaining of being sick.

Interviewer: Before you knew about your sickness?

Interviewee: Yes.

Interviewer: Alright.

Interviewee: Because, after I have told him he then suggested that we must start using a condom and then I said it is fine, I do not have a problem with that too.

Interviewer: Okay.

Interviewee: And then after that, he was always complaining about feeling some heat and I encouraged him to go to the clinic.

Interviewer: Feeling some heat as in how?

Interviewee: He was feeling hot.

Interviewer: Like was he feeling hot or it was a certain sickness?

Interviewee: I do not know how it was, but he used to say he was feeling some heat (oh, okay), he used to cough and had flue (yah), so I suggested that he must go to clinic and he refused (yah) and he said he would go to pharmacy to get some pills and I said okay it’s fine (yah). Ey, after that then, since I have broken up with him, I do not about this thing…oh, he then said…he told me he was at the clinic, and I said wow! What are you going to do there (yah), he then said he had a problem, he had just had a problem his-his thing (penis) is painful

Interviewer: As in his genital part?

Interviewee: Yes (okay), private part, and then I said to him, but I know (mmm), he said how do I know? I said I know that you have slept with another girl and you didn’t use a condom (mmm) he asked what do I mean and I told him I know that (yah), trust me (mmm), then he said he doesn’t know what I am talking about, and the I said I hope you are not lying that you are at the clinic right now (ummm). When we met again, we were so chilled, and we were talking and then I asked him to tell me the truth if he has really slept with another girl without using protection, and he admitted that e as done that (yah) and he promised not to ever do it again because he has learnt his lesson. Then I asked him if he was aware that he is the one who as infected me with the virus (mmm), because he has never-he never wanted us to talk about this thing (yah), when I talked about it, he would say why are we talking about this, don’t we have other things we can talk about (mmm), so I asked him if he is aware that he had infected me with this thing.

Interviewer: I would like to ask whether it was before or after you knew about your life status when you found out that he slept with another girl?

Interviewee: It was after?

Interviewer: But you were somehow suspecting him?

Interviewee: Yes, I was suspecting him.

Interviewer: Because he used to get sick now and then before you knew about your status?

Interviewee: Yes, he used to get sick a lot.

Interviewer: Okay.

Interviewee: So, then I asked him if he is really aware that he was sick, but he doesn’t want to take care of himself (mmm, mmm), and you are the one who infected me but you do not want to talk about it, instead you are ignoring (yah), then he said “is it me now?” and then I asked him if I have ever lied to him before, because I have never been dishonest with him (yah), and I also asked if I have ever told him that I was sick before (yah), because after sleeping with him I have told him that I was sick and I did not hid that from him (mmm).

Interviewer: And it was your first time?

Interviewee: First time.

Interviewer: Getting infected by this virus?

Interviewee: Yes.

Interviewer: And having sex as well?

Interviewee: You see that thing?

Interviewer: Yes.

Interviewee: So, he just got irritated, he got irritated and you could see that he was irritated and the he stopped talking to me on WhatsApp.

Interviewer: May I…okay may I cut your conversation, so before you guys started having sex, were you guys been talking about testing and everything, like asking about each other’s status and disclosing that?

Interviewee: Before?

Interviewer: Yes, like telling him that you were HIV free

Interviewee: I do not want to lie

Interviewer: And wanted to know his status too?

Interviewee: Let me think how old I was then…I was 17

Interviewer: 17 years old?

Interviewee: I was 17 years old and he was my first boyfriend.

Interviewer: So, you were not thinking about all of these things?

Interviewee: I have never thought about the, I do not want to lie

Interviewer: Yah, yah, yah, I understand.

Interviewee: I have never

Interviewer: So, you have never talked about sex and testing everything before, like everything just happened?

Interviewee: Yes, it just happened

Interviewer: Yah.

Interviewee: It just happened.

Interviewer: Even though you used to see him doing all those hide and seek games and you were somehow suspecting that he is a two timer, but you just ignored that?

Interviewee: Yes, I wasn’t pay much attention on that, I did not even think about doing any HIV testing with him (yah), and I always regret that if I paid attention to his behavior and took some steps

Interviewer: Even when you saw him sick, nothing came into your mind about taking healthy actions about that

Interviewee: He was not getting so much sick

Interviewer: But now you can see that-that

Interviewee: Yes, now I can realize that maybe it is because of this sickness he was hiding from me.

Interviewer: Sorry, you may continue, I am sorry to interject while you are talking.

Interviewee: No, feel free, there is no problem. So, yes, it was something like that (yah), and then I told him that he is the one who infected me with this virus (mmm) and then he said he doesn’t know what I am talking about (mmm) and he doesn’t want to talk about it. Then I said, what makes you don’t want to talk about it is that you are aware that I am telling you the truth now (mmm), and the he requested that we changed the topic and I said to him let us talk about this thing because you are busy infecting other people out there (mmm, yah), and now he is spreading to other women, so I don’t know maybe I was saying this because it my mind there is that thing he is aware that he has the virus (yah) and he has that attitude that if you have unprotected sex with an HIV negative woman you will get rid of the virus

Interviewer: Are there any people who believe in that thing?

Interviewee: Yes, they are and that what came to my mind when he came from the clinic and told me that he had sex without a condom and I told him to stop infecting other girls (mmm), so that is where he stopped talking with me

Interviewer: So, that is where you guys started fighting with each other?

Interviewee: Yes, it has been five months now

Interviewer: Yes, I understand. So, do you think how your body looks affect the way others look reject or accept you? I mean the way your body looks.

Interviewee: Em….. (Laughing)

Interviewer: Do you think people accept you maybe?

Interviewee: To be honest, (mmm), they accept me (mmm), it’s just that…what can I say though, I am that kind of a girl who-who…what can I say, I have a lot of friends (okay), and I was so closed to them (yah), so they do not have a problem (yah) they do not have a problem, even when I was going with them, I felt comfortable (mm) I was okay and I was so full of myself and they used to tell me that I should stop complaining about my height because they do not see me that way

Interviewer: Yah, it’s what they like

Interviewee: Yes, and they say they really like that I am tall, and they wish they were taller like me (yah), so I didn’t have any problem and I could feel myself when I was with them (mmm), we were in the same class, doing same subjects (yah) and it was okay. However, for some people it was not the same.

Interviewer: How, did they reject you?

Interviewee: Yes, I can say something like that perhaps.

Interviewer: What makes you feel that people reject you because of the way your body looks?

Interviewee: Eish, just...there is a time where you feel like a person is…okay I just take is as that thing of fitting in you see (mmm), so you will just be aware that you do not fit to these kind of people (mmm, laughter), also because of your height (mmm), like you can see that these people are curved, those girls who wear short outfits (mmm), so even if you can attempt to join them you can just see that you do not fit into them (laughter), you do not fit at all.

Interviewer: You do not fit there

Interviewee: Yes, things like that.

Interviewer: So, do you feel like you are being rejected by those people or you just see it by yourself that you do not fit there?

Interviewee: No no no! You can just see it by yourself (laughter), at times people will laugh at you when you are showing up into them, and so you will just think the worst

Interviewer: So, they react in a way that you end up asking yourself what’s wrong with you.

Interviewee: Yes, you end up thinking that maybe it is my height that makes them funny (yah) why are they laughing though (yah) and I be like, yes maybe it is my height that is funny to them, so I always assume it is my height then (yah, I understand you).

Interviewer: So, how does positive or negative feedback form significant others on your body appearance makes you feel.

Interviewee: (Laughter)

Interviewer: Maybe you can start with positive feedback about your body, like how does your body appearance to them makes you feel.

Interviewee: Ey…ay (laughter).

Interviewer: No, feel free to talk.

Interviewee: Ummm…. I feel happy.

Interviewer: You feel happy?

Interviewee: Yes, I don’t want to lie, I feel happy (yah) because there is that thing that at least there are some people who likes what I don’t like about my body (yah), you see that thing (yah), so I feel so happy that at least some envy this thing I don’t want (mmm), some even say to me they wish we could exchange our bodies because they do not like the fact they are old but they look young, and that they wish there were me, and I say to them too also wish to be like them and that really makes me feel happy (mmm), so I like their opinions (yes, I can see).

Interviewer: What about negative feedback then?

Interviewee: Ey…(laughter) that one doesn’t sit well with me (laughter)

Interviewer: How?

Interviewee: Ey…just! You know those kind of people who give me negative feedback do not please me, because I have a low self-esteem (mmm), I still have that thing you see (mmm)

Interviewer: So, Yah, it makes you feel…

Interviewee: They just fulfill that bad feeling I have about my height, and then wish I was shorter (mmm), I even wish I could change somethings in my body because I am ashamed of myself. What can I say, I can talk with you, but it takes time for me to become too close to a person (mmm), because I am too shy (yah,yah). I also do not like to be in limelight because of my height perhaps, in such a way that if I have been appointed to say something or… I was doing drama at school (mmm), so if I had to say something it was hard for me because I knew that people would be looking at me, you see (eh) and people will be making fun of me that “hey, this one is still in school, yoh she is too old, why can’t she finish school”, because of my height.

Interviewer: But you know that you are young.

Interviewee: I know that I am young (mmm), I know that, but the problem is my height that is not compatible with my height (laughter) yah. (Laughter) So, that was a confusion and you could see that it was really confusing, and you don’t like-you don’t like standing in front of other people, I do not like it (mmm), but once I am in front of people I can talk, but…ah

Interviewer: Because you are always thinking that people will judge you about your body

Interviewee: Yes, what are they going to say about my body, yes-yes (yah) I think about that thing, that they will make funny comments about my body, and I sometimes wish that if I had a little bit bigger booty (laughter), you see (laughter), so that I cannot just be straight girl with no shape (laughter) eish, mxm, you know girl (laughter) (yah) (laughter).

Interviewer: I understand, so what is the importance of having a big booty?

Interviewee: (Laughter)

Interviewer: Because you have a beautiful body.

Interviewee: No! I disagree! I do like myself, I do not want to lie, and I like myself (laughter) (eh), the problem is when a person says she/he is seeing a model from me, and I do not know-I do not know what happened, because I used to very active (mmm) I was…you see, even at school I was always participating in sports activities (mmm), but now, I do not want to lie, I am lacking a lot and I can even realize that (mmm), even if I try, I can try but I can just see that no ways, it is no longer the same again (laughter).

Interviewer: What is it that you think it has made you not become active as you used to be before? Or is it because of your health status that has made you feel like ah…

Interviewee: My health-my health status…ey I do not know…I do not-I do not have a pro…

Interviewer: Did your health status influenced you to stop…

Interviewee: I do not have a problem with it.

Interviewer: But…

Interviewee: But when I think about it, I think so deeply (eh) and I will be like oh Lord (mmm), especially when I have to come to the clinic (mmm), yoh! I do not want to lie…

Interviewer: It doesn’t feel good.

Interviewee: It doesn’t feel good, I drag myself (mm) especially when I have to do blood tests and all the staff, I drag myself (mmm) and tell myself that you see today, you see today (eh) and I can feel that I am really not feeling okay. I do not know, it’s like-like, I do not know whether I should talk or what (mmm), I do not know, I do not know, but I have told myself (mmm), but I do not know, I do not know how I can explain it, because now I realize that this thing…and my mother encourages me and say “go and join the support groups where you can find some motivation (mmm) and talk with your peers (mmm)you will be okay and your mind will be opened” (mmm), you see that thing? (mmm), she says “they will enrich your mind, instead of sitting alone here at home, overthinking because now you are different”, and it is true I am no like those people, because there are things that I cannot do while other people can do (mmm), and the fact that there are things that I can’t do while others can doesn’t make me feel bad (mmm) you see, because I am not as active as I used to be.

Interviewer: As you used to be before?

Interviewee: Yes.

Interviewer: So, does the way your body looks limits you in initiating and maintaining relationships with significant others, like a partner or a friend?

Interviewee: (Mumbling….silence)

Interviewer: Or can I elaborate this question further?

Interviewee: Yes, please explain to me.

Interviewer: I mean, doe the way your body look limits you in initiating friendships

Interviewee: With other people?

Interviewer: With anyone, someone who is significant, to initiate a relationship and also to maintain that friendship you have initiated in that relationship, maybe it can be your romantic partner, can be a friend or anyone, because of the way your body looks and everything about you in general.

Interviewee: (Laughter)

Interviewer: For example, maybe it can make you say or feel like because you are like this I cannot have or make friends, I cannot try to get closer to people wanting to become friends with them, and again, when you already have a friend

Interviewee: Yes, it happens (yes), that thing happens (mmm), it happens and it can just be clear to yourself that you see in these group of people (mmm), I will, definitely I will feel isolated (mmm), and I sometimes realize and you can just observe people (mmm), you can even look at the person’s body structure, you can even see a guy and fell that this is my crush (laughter), but I am not in his standard (laughter) (eh), especially with the body structure (eh), so you can see that as much as he is a guy and you can see he is the right guy, but you can just see that you do not fit and have that girl in mind who can be his perfect match (eh). So, you can see that this kind of guy may like tall girls, but he may prefer those ones who have a good body structure (mmm), a girl that have small boobs, curved (mmm) and beautiful, so that thing happens (okay, okay).

Interviewer: So, let us say you have already initiated friendships, so does the way your body looks enables you to maintain that friendship, or at times you feel like, eish yah, we are friends, but I do not feel like sustaining this relationship, do you ever experience such thing?

Interviewee: Ey…. (Silence)

Interviewer: It doesn’t happen?

Interviewee: You mean due to my body?

Interviewer: Yes, about your body…

Interviewee: No, it doesn’t happen.

Interviewer: It doesn’t happen?

Interviewee: No, my friends um-um, what can I say, those one who are at home are very few (mmm), what can I say, I am the only one amongst them who is a little bit bigger and taller (mmm, okay), so they have tiny bodies and the fact that they also hate being big, so sometimes I see myself having a fat body than them (mmm), I see myself being too fat because they do not like, what can I say, they work out on their bodies a lot (oh, okay), things like that and one of them is doing modelling (okay), so you can see they do not want fat bodies, so this makes me feel comfortable around them sometimes (okay), I just ignore my body size and feel comfortable (mmm, okay)

Interviewer: So, if I can ask about your health status, does it limits you from initiating relationships, for instance, if a person is approaching you, be it a friend or a guy, does it makes you feel uncomfortable being in a relationship with that person because of your health status?

Interviewee: It happens psychologically (laughter)

Interviewer: Yah, you find that even if you…

Interviewee: In your mind (laughter), what can I say (mmm) honestly speaking, umm-umm…what can I say, this thing comes to my mind (mmm), and maybe when I am thinking about a guy (mmm), what I can I say, so far the guys who are approaching me are-are not

Interviewer: Are not pleasing you

Interviewee: Yes, I am not interested in them, because they do not have the qualities I need from a guy (okay), or let me say in the standard I have designed for myself (umm), but if I look at a person, if I look at a boy, if I just look in general, maybe a guy that I am crushing on (umm) I will be like oh Lord, so it is a good but the problem is what am I going to say to him if we end up becoming an item because I like being honest (umm), you see that thing (yah), I like being honest, I-I prefer being honest, you see that thing (mmm), so I always ask myself, how am I going to explain my health status to that person (mmm), because even though a crush doesn’t last, but it is obvious that the day I will tell him about my health status, definitely he will leave me

Interviewer: He will leave

Interviewee: Yes, so you see that thing? (Yes), so I always think about that. I do not want to lie, I do not have a boyfriend

Interviewer: You do not have a boyfriend

Interviewee: Yes, because I have my own reasons (yes, because..) now I want to focus a little bit in my life, until I am okay, so there is that thing, there is that thing

Interviewer: Until you are okay like how?

Interviewee: Maybe financially wise

Interviewer: Financially wise?

Interviewee: Yes

Interviewer: So you think that once you are financially stable, it would be easier for people to accept perhaps? Or you think it can be easier for you to initiate relationships, or it can be easier to explain to people about your health status too?

Interviewee: I think so, it’s just that eish, I am just thinking, I do not want to lie, I am thinking (mmm), I think it can be easier when I a financially stable, because I am simple person, I do not like a lot of things (mmm), I do not like a lot of things (mmm), so maybe I can focus on something (mmm), let me say perhaps I can have a business or something (yah), you see maybe I will be working, so you see maybe I can focus more on that thing (mmm).

Interviewer: Don’t you think that maybe, when you are financially stable, people can accept you even if you can disclose your health status due to the fact that you will be moneyed?

Interviewee: (Laughter) I do not want to lie, I do not know about that. According to my own knowledge, this thing, I do not know whether I will give you the correct response (yah), but according to my personal view, I mean that having money can help take care of myself better (ow) or more than I am taking care of myself now, can you see that thing? (Oh yah), maybe it can help practice healthy behaviors (yes) you see

Interviewer: And be able to love yourself more

Interviewee: Yes, and loving myself, you see that thing, and get something that will satisfy me (mmm), you see that thing (mmm), because at times when I have some flue or anything that can happen (mmm) I panic a lot over nothing (mmm) and wish if I had these pills, what do they call them? This thing, these pills, what do they call them…umm I forgot what do they call it…the one for boosting (ow), those one for boosting (ow) that helps you gain more strength (yes), so I will just wish that I would be taking those pills (mmm), so I think it is money (mmm), so I do not know how it works in males (how it works, mmm)

Interviewer: So, I do not know whether I got you right here, so you mean that for now, as you have been mentioning earlier that even now you do not trust yourself, you do not see yourself as beautiful as much (mm) (background noise), so you think that when you are financially stables, you can be able to take care of yourself and boost your confidence, and you can also be able to initiate relationships and you can feel like people can accept you?

Interviewee: Yes, accepting yourself starts from within you (ow), but I am thinking that maybe if I had something that pleases me (yah) you see that thing (yah), something that will free-that will free…that will make your heart feel happy

Interviewer: Within you first

Interviewee: Within me (yes), by telling yourself that I am okay now, at least I have this and that (yes), my mind will start telling me that you see now, you are okay now (mmm), you see now, you are okay (yah, I get you.)

Interviewer: So, do you think that significant others influence how satisfied you are with your body?

Interviewee: (Silence…..laughter……background noise) Not exactly

Interviewer: Not exactly (Laughter) Why?

Interviewee: (Silence….background noise….laughter) is this question almost the same with the first one on how people (yah)

Interviewer: Like people who are significant in your life, you can even talk about your boyfriend that you had, like the way they-they…like is their influence makes you satisfied with your body.

Interviewee: Before

Interviewer: Before?

Interviewee: Yes, when I was in love with him (yes), he wasn’t seeing anything wrong with me

Interviewer: So, you are saying before you were what?

Interviewee: I was satisfied

Interviewer: Oh, you were satisfied

Interviewee: Yes.

Interviewer: Was he influencing you to be satisfied?

Interviewee: Yes, because he realized that I was lacking some self-esteem (yes), so he had no problem with my body (yes), so you see, and another thing maybe due to the fact that he knows me (yes). You know what more problematic (yes) is that before I got infected (mmm), I was-I do not want to lie, I was okay because I was wearing size 38 (mmm) I had a rich body, I was full, so now that I am losing weight, I think it’s because I keep on thinking of getting back to my old weight-being fat again (yes), I want to be my old self (yes), so he knows me in my old rich body (yes), so he had that thing-he had thing and he kept on comforting me that I am okay (you are okay) and when I look at myself, I could see that I have lost weight (mmm) and he could say that is not true, it’s because you have constructed it in your kind, but to me you are okay, you do not have any problem (wow!). so that was that thing, that I really wanted to gain weight because I remember myself when I was fresh and big (mmm), so you see when I am like this, my height, oh sorry, my body (mmm) and my height doesn’t correlate so you see that thing (mmm).

Interviewer: Yes, I get you. Can I please go back a little bit, as you have mentioned that earlier that there was someone you were in love with, and later you found out about this, so did he do an HIV testing?

Interviewee: No

Interviewer: So he has never tested until you guys broke up?

Interviewee: He has never done an HIV testing and he has never

Interviewer: Have you tried to convince him about testing?

Interviewee: I did all in my power, I have done everything (background noise), because even here, when I first came here, there was sister Mkhize (mmm) who insisted my partner should come too, and I told him (mmm), so he promised to come do a test here in the clinic (mmm), because he doesn’t want to test from local clinics at home (mmm). I was staying here, because I was studying around here (mmm) so clinics from home were far from me by that time (mmm), so I asked him to at least come and do testing even when he is around here

Interviewer: So, you wanted you guys to do it together, or you wanted him to do it alone?

Interviewee: I wanted him to do it alone

Interviewer: Okay

Interviewee: I already had it by that time, when I felt that I was getting weak (ow), so I insisted that he must go and do it, and he refused.

Interviewer: He refused?

Interviewee: He agreed that he will come, so I keep on begging him and he kept on refusing, so I ended up saying, go and do it anywhere (anywhere) because you do not spend a lot of time here, so just go anywhere (yes, so that you will know at least) and he refused

Interviewer: So, after you have found out about your HIV, he said you guys will be using protection from then onwards, so were you guys maintaining that practice, like were you always using a protection?

Interviewee: I do not want to lie, we were always using protection (oh, okay)

Interviewer: He had no problem with it?

Interviewee: He was the one who was encouraging it, because what I know is that if you are taking HIV treatment (mmm) while the other person is not eating it (mmm) I do not know who will get sicker, either me or him (yah) is it him?

Interviewer: I am not sure about that.

Interviewee: You seen that thing (yes, yah), so that is how I explained to him and he is a coward (mmm) because using a protection is the right thing

Interviewer: Yes, and you were also protecting him

Interviewee: Yes

Interviewer: And yourself too

Interviewee: Yes.

Interviewer: Okay, we are about to finish. So the last part of our questions will be talking about resilience, as you have mentioned that people have funny comments and mention different things about your body and everything, so this questions will be asking at how you negotiate a positive body image, like despite all the negativities you are encountering about your body, how do you maintain a positive attitude towards your body image. So how do you manage to accept your body as it is? What do you do to accept your body the way it is?

Interviewee: I just tell myself that there is no surgery heart, (mmm), that is what I am telling myself (mmm) so even if I become okay, I cannot change how I look, this how God has created me (mmm), God has created me like this (mmm), which means that at least (laughter) I do not know how to explain this, because at times, don’t take wrong, but at times I compare and say if I was too short perhaps (mmm), would I be loving myself or (background noise).

Interviewer: Pardon?

Interviewee: Would I have been loving myself or I would be complaining too? (yah) and I would be complaining again, so sometimes I accept-I accept myself, especially when I have worn something that looks good on me, I tell myself that today I love myself and I thank God that he created me this way (mmm), so I do not want-I do not want to doubt God (mmm), because he has created me to become something else, I would be complaining about that thing too (mmm), would I be accepting that thing? (I see). As much as we are always complaining to God, sometimes we must thank him (yah), so I thank him for that, and you know what I just tell myself that there is nothing I can do about my height (mmm), I am tall, and we are all tall at home (yah), therefore I will accept myself

Interviewer: It is not like there is something wrong with me

Interviewee: Yes, you see that thing? (Mmm, mmm, yah)

Interviewer: So, if people are making hurtful remarks about your body, how do you manage with that thing?

Interviewee: (Laughter) Ey, I become furious

Interviewer: You are getting what?

Interviewee: I become furious, and before I used to be aggressive

Interviewer: You become aggressive?

Interviewee: Yes, I become aggressive, and I get irritated, I get angry (yah, yah), I get annoyed. So you see, I just ask one question that is it your first time to see a tall person (mmm), especially when you are also staying here at Mayville and have the audacity to tell me that I am tall (mmm) I will ask if you have ever seen me short, because I have been tall since I was young (mmm). that thing irritates me, it annoys me (mmm) because I am also aware that I am tall (mmm), so there is no other thing you can say perhaps, because of constantly reminding me that I am tall, so there will always be those who will be making nasty comments (mmm), and you may find that I am feeling myself and a person will make fun of me, ey, you see (yah) because even now I have not totally accepted how my body looks (mm)

Interviewer: So, after…since you have mentioned that at times you become aggressive and revengeful or talk to that person, so when you are alone thinking, how do you manage to deal with all the thoughts of what people are saying about your body?

Interviewee: What is better to, something that is better to me is to open up (mmm), because once I have opened up to a person (yes) I do not want to lie, that issue get out of my mind (yes), I just let it go off my mind (mmm) it become easier to get rid of it (yes), but if you have done something wrong to me and I fail to open up to you, I will just ignore you

Interviewer: You do not feel comfortable

Interviewee: I will be pretending, yes I can talk with you (mmm) but it will be just a pretense until I tell you that you know what, I didn’t like this and that (mmm). So, I will be doing that because I am avoiding any tension between me and you (yah), so I will prefer we talk about it, because I do not like to hold grudges or to have someone that I do not talk to (yah), so I will tell you that tone and two didn’t sit well with me (mmm), I didn’t like it (mmm), so, if had opened up about something it becomes better, but if I am keeping it to myself, let me say if you has said something bitter to me and I decided not to say anything but to just leave you like that, that thing irritates me (mmm, yah), but I will come back to you and disclose that what you have said irritated me but I want us to talk, because I will be ignoring you and do not want to talk to you (mmm) over a little thing (a little), maybe I took it seriously (mmm). So, talking to someone can make me feel better (mmm)

Interviewer: I get you, I get you. So, once you have opened up about that issue you feel better afterwards?

Interviewee: Yes, I feel okay and I can talk to my mother.

Interviewer: So, do you have any other…oh you have mentioned that it is your mother (laughter), so do you have any people in your life that listens to you, like people who listens to you when you are talking, people who give you support whenever you need it?

Interviewee: Yes, it is my mother

Interviewer: Why?

Interviewee: (Background noise)

Interviewer: Please try to speak a little bit higher

Interviewee: I am always talking to my mother (eh), I talk to my mom a lot (eh), because I can to her about everything (mmm, mmm, mmm) you see things like that. So I can even tell her that I am panicking even if a silly thing has happened (mmm), so what makes me talk to her is that she can take some actions (mmm). For example if I tell her that mom I am not feeling myself, I am losing appetite, I do not like food (mmm), she can try to help me, do you see that thing (ow), so even if I feel like I have lost weight, I can also talk to her that no mom, it seems like I am losing weight, and she will say “this thing is in your mind (yes), you are just telling yourself that you have lost weight, and you are the one who knows that you have lost weight” and you may find that I may see myself that I have lost weight, but when I wear something I will see that it is still the same

Interviewer: It is still tight on you

Interviewee: Yes, you see that thing (eh), so she is the person I can talk to her because she always try to help me.

Interviewer: So, were you able to talk to your boyfriend, like when you had a problem, were you able to tell him, was he supportive or?

Interviewee: To be honest, he was supportive, he was really supporting me shame (yah), and I was able to talk to him, there was nothing that I was failing to talk to him about (yah) even if I had lost appetite and couldn’t eat he would suggest that I eat this and that (mmm), if certain something happens, he would tell me what to do, if I am failing to do it he will be encouraging me to keep on trying, and he was also recommending a clinic even though I have never heard him mentioning going to clinic

Interviewer: But he was supportive to you?

Interviewee: Yes, he was encouraging me to go to the clinic, if I wasn’t feeling okay, he would tell me that I will be fine. I had that thing you know, I do not know what was happening, but I was overthinking and feel confused (mmm, mmm), so he would sit me down and made sure that he says something, so he would tell me to stop overthinking, you see (mmm), you see things like that (yah)

Interviewer: I get you sister, I do not know-but we are done, so I want to ask if you have any questions for me, or any question you have or any suggestion you may like to add, anything you may like to add in everything we have just talked about.

Interviewee: In everything we have talked about

Interviewer: Yes, like anything you may like to say, be it a question or…

Interviewee: Oh okay! My question for you is that, as you have mentioned about your height earlier (mmm) it is something-oh I know that you can accept something into your heart (yah), how do you convince your heart that whatever you are telling yourself is exactly what it is?

Interviewer: how do I convince myself?

Interviewee: Yes

Interviewer: As you have mentioned earlier that at times we have to appreciate the way God has created us (mmm), so at times it doesn’t feel good for me that I am short (mmm) because at times I do not get the respect I deserve from people (yah), because… for example, I am older than you, but I know that you cannot give me much respect because you will be seeing a child from me. So, I ended up learning to accept myself, and workout on myself, as I have mentioned that I am a little bit shy, so instead of blaming God I must thank God for what he has given me and workout on what he has given me, and I should try not to be shy, because what I also understand is that God does things for a reason (yah), its God plan, so I-I sometimes have that hope that there might some opportunities that I might get in life because of my height, just like you, because for me it is an advantage that because there are lot of things that you can achieve, especially career wise, there are lot of jobs that you can get due to your height. So, something of the things lie-I have also learnt to accept myself, even though it was not easy (mmm), yes it was not easy, but I have learnt to accept that this is who I am, because what we must know about life is that there are things that we cannot change, but the more you accept it the more you feel better. So, I have learnt to accept and love myself (mmm) because this is who I am and there is no way I can change that. So, I am always telling myself that I am beautiful, even though when I look at myself at times, I can feel that I am not okay. Since we are ladies, there are kinds of clothes that I cannot wear because of my body structure, so there are things like long dresses especially the traditional attires, you know those beautiful clothes (yes) I cannot wear those dresses because it doesn’t fit well with my body. I like them, as you have also mentioned that you like shorts and you wish to wear them, but you cannot, so at times, you just have to tell yourself that this is who you are, even though people gave a tendency of making funny comments about our bodies that we do not like (mm), at times you will end up crying, but you end up telling yourself that this is what it is. As I grew up I had that hope that I was going to get tall, I would be tall, but there was point whereby I accepted that this is the endpoint for me (laughter). I do not know whether I have answered your question well?

Interviewee: Yes, you responded very well. So, for being shy how did you work out on that because I am too shy?

Interviewer: Eish

Interviewee: I do not like something on lime light or spotlight especially that will force me to talk, because I can talk (yes, but…) you see now I can talk to kids because I am teaching kids at church

Interviewer: Ow, you are a Sunday school teacher?

Interviewee: Yes, but my problem is that I can stand in front of the kids, but I can be in a situation that I will have to talk to elders, I can’t.

Interviewer: Yes, it is not easy, so what has happened to me, since I have told you that I am old, I have realized that people do not give me the respect I deserve and I have realized that I am the one who is allowing people to do that because I always tell myself that I am shy blablabla and I have missed many opportunities in life, and there are lot of things that I have limited myself because I used to tell myself that I can’t go for this because I am shy, so I have realized that there are lot of things I am missing and my life is stagnant, so at times it’s good to get out of the box and try your level best, even though it is not easy. So I always try to be strong, even if we are doing presentations I ask to present, and I know it is not easier, but you have to try hard, you see (mm) tell yourself that I have to do it. Right now I am working on it, and hardly working on it because I can realize-like at times I ask myself that my peers are far beyond me in life, but we all started in the same pace and when you do an introspection you can see that it is because I have been limiting myself by telling myself that I am short, I am shy. Another thing I have learnt in life is that you are not supposed to accept bad things in life (mmm), but you must always try to change that thing. You must be grateful that you have managed to realize that this thing is bad, so do not nurse it, but try to make good thing out of it. So this is life, and another thing I have told myself in life is that no one will ever work for what is mine or I supposed to work on, no one will ever change my height or make me not shy unless I stand up for myself, so wherever there is a will, there is always a way. So that is how I’m-I am still in the process of changing my life though. Like I am trying.

Interviewee: You are trying (laughter)

Interviewer: I am trying (yes), I am not perfect but I have realized that I have no choice, so I will have to do it, because life has to go on (ey) I can’t keep on blaming God or whatever. At home we are not too tall, but I am the shortest, and I am the first born so (wooow), so you know like-but it is fine, this is how it is. They are short people from my extended family, nut I am the shortest in my family, like the shortest one (laughter) yah (laughter). I do not know if you have anything you may like to add or ask again

Interviewee: No

Interviewer: You are okay?

Interviewee: Yes

Interviewer: Thank you so much Wendy, that was so informative

Interviewee: Yes

Interviewer: (Laughter)
