# Supplementary material for: “I like the way I am, but I feel like I could get a little bit bigger”: Perceptions of body image among adolescents and youth living with HIV in Durban, South Africa
Source: PLoS One. 2020 Jan 10;15(1):e0227583. doi: 10.1371/journal.pone.0227583 (PMC6953798; doi:10.1371/journal.pone.0227583)
Supplement: S2 Appendix — (DOCX) [file pone.0227583.s002.docx]

Thoughts, feelings and experiences of AYPLHIV on the appearance of their body were examined by probing the following:

**APPENDIX 1: INTERVIEW SCHEDULE**

- What comes to mind when you think of “body image”?
- How would you describe the way your body looks?
- How does your perception of your body image affect you? Think mentally, physically

and emotionally?

- In what ways does the way others perceive you affect your mood?
- How does positive or negative feedback from significant others on body appearance make you feel.
- In what ways does body appearance influence acceptance/rejection by peers?
- Has your body image ever stopped you from doing something? If yes, in what ways?
- In what ways has your body image affected your social life? Has it affected the friends you hang out with? Your dating life? If so in what ways?
- In what ways has your family impacted your perception of your body image?
- Would you say that you are satisfied with the way your body looks? Explain.
- Thinking of the issues we have discussed related to body image, how do you copy with the various challenges you face related to body image and living with HIV/AIDS (Probe on inner strengths, interpersonal relationships and external support networks).
